# Habitat segregation and ecological character displacement in cryptic African malaria mosquitoes

**DOI:** 10.1111/eva.12242

**Published:** 2015-03-08

**Authors:** Billy Tene Fossog, Diego Ayala, Pelayo Acevedo, Pierre Kengne, Ignacio Ngomo Abeso Mebuy, Boris Makanga, Julie Magnus, Parfait Awono-Ambene, Flobert Njiokou, Marco Pombi, Christophe Antonio-Nkondjio, Christophe Paupy, Nora J Besansky, Carlo Costantini

**Affiliations:** 1 Institut de Recherche pour le Développement (IRD), UMR MIVEGEC (UM1, UM2, CNRS 5290, IRD 224)Montpellier, France; 2 Laboratoire de Recherche sur le Paludisme, Organisation de Coordination pour la lutte contre les Endémies en Afrique Centrale (OCEAC)Yaoundé, Cameroon; 3 Department of Animal Biology, Faculty of Sciences, University of Yaoundé IYaoundé, Cameroon; 4 Eck Institute for Global Health & Department of Biological Sciences, University of Notre DameNotre Dame, IN, USA; 5 Centre International de Recherches Médicales de Franceville (CIRMF)Franceville, Gabon; 6 SaBio, Instituto de Investigación en Recursos Cinegéticos (IREC), CSIC-UCLM-JCCMCiudad Real, Spain; 7 Faculty of Medicine, Universidad Nacional de Guinea Ecuatorial (UNGE)Bata, Equatorial Guinea; 8 Institut de Recherche en Ecologie Tropicale (IRET)Libreville, Gabon; 9 Sezione di Parassitologia, Dipartimento di Sanità Pubblica e Malattie Infettive, Università di Roma ‘La Sapienza’Rome, Italy

**Keywords:** *Anopheles coluzzii*, *Anopheles gambiae*, cryptic species, ecological character displacement, ecological speciation, habitat segregation, malaria vector, molecular forms, niche partitioning, saltwater tolerance, spatial ecology, species distribution modelling, urban pollution

## Abstract

Understanding how divergent selection generates adaptive phenotypic and population diversification provides a mechanistic explanation of speciation in recently separated species pairs. Towards this goal, we sought ecological gradients of divergence between the cryptic malaria vectors *Anopheles coluzzii* and *An. gambiae* and then looked for a physiological trait that may underlie such divergence. Using a large set of occurrence records and eco-geographic information, we built a distribution model to predict the predominance of the two species across their range of sympatry. Our model predicts two novel gradients along which the species segregate: distance from the coastline and altitude. *Anopheles coluzzii* showed a ‘bimodal’ distribution, predominating in xeric West African savannas and along the western coastal fringe of Africa. To test whether differences in salinity tolerance underlie this habitat segregation, we assessed the acute dose–mortality response to salinity of thirty-two larval populations from Central Africa. In agreement with its coastal predominance, *Anopheles coluzzii* was overall more tolerant than *An. gambiae*. Salinity tolerance of both species, however, converged in urban localities, presumably reflecting an adaptive response to osmotic stress from anthropogenic pollutants. When comparing degree of tolerance in conjunction with levels of syntopy, we found evidence of character displacement in this trait.

## Introduction

One of the major interests of evolutionary ecology is understanding the ecological forces that are responsible for the evolution and coexistence of closely related sympatric species. Cryptic species are morphologically indistinguishable and often very closely related genetically and ecologically. Increasing application of DNA sequence analysis has accelerated discovery of cryptic species and revealed their abundance across taxonomic groups and biogeographic regions (Bickford et al. 2007; Pfenninger and Schwenk 2007). Despite theoretical developments, however, empirical genetic and ecological investigations have yet to provide a detailed understanding of the mechanisms responsible for the build-up of ecological and reproductive barriers during ongoing speciation (Sobel et al. 2010; Nosil 2012). To the extent that seemingly identical, recently diverged or emerging species exploit the same limiting resource, classical ecological theory predicts that competition will prevent their stable spatial coexistence. As such, the long-term sympatric coexistence of cryptic species implies the presence of intrinsic biological differences that facilitate specialization and ecological niche partitioning, reducing the strength of interspecific competition (Szilagyi and Meszena 2009; Sobel et al. 2010; Nosil 2012). Uncovering the nature of these differences is a fundamental goal in studies of speciation, but also is critically important to biodiversity assessment, conservation efforts and control of economically and medically important insect pests (Coluzzi 1970; Ferguson et al. 2010).

Particularly well-known examples of cryptic diversity occur in the mosquito genus *Anopheles*, which contains the vectors of human malaria parasites (Coluzzi 1970). Nearly all *Anopheles* vector species are members of cryptic species complexes (Harbach 2013). By far the best studied is the group of Afrotropical species known as the *Anopheles gambiae* complex, containing the vectors responsible for the majority of malaria transmission in sub-Saharan Africa. All eight currently recognized species in this complex (Coluzzi et al. 2002; Coetzee et al. 2013) are morphologically indistinguishable, yet differ not only in terms of their contribution to malaria transmission but also in ecological characteristics, notably their trophic habits and aquatic larval habitats (Coluzzi et al. 1979; White et al. 2011b). Three species with nonoverlapping distributions are saltwater tolerant, breeding in brackish water along the coastline and associated islands of Western or Eastern Africa (*An. melas* and *An. merus*, respectively), or in geothermal springs of high mineral content in the Semliki forest of Uganda (*An. bwambae*). The distribution of each of the salt-tolerant species overlaps that of at least one of the obligate freshwater species in the complex, suggesting that tolerance to saltwater may have played a role in ecological diversification of this species group.

One of the most recent examples of cryptic diversification in the *An. gambiae* complex is occurring in the nominal species and most important malaria vector, *An. gambiae*, a freshwater taxon whose geographic range spans most of tropical Africa (Coetzee et al. 1993; Sinka et al. 2010). *Anopheles gambiae* carries extensive inversion polymorphisms on chromosome 2, which may contribute to ecological segregation of populations (Coluzzi 1982; Coluzzi et al. 2002; Manoukis et al. 2008; Cheng et al. 2012). However, genetic evidence independent of chromosomal inversions supported the subdivision of *An. gambiae* into two assortatively mating cryptic lineages provisionally referred to as the M and S molecular forms, whose distributions overlap in West and Central Africa. Premating reproductive isolation – although significant (Tripet et al. 2001; Diabate et al. 2006, 2009; Pennetier et al. 2010; Dabire et al. 2013; Sawadogo et al. 2014) – is incomplete, and there is no indication of intrinsic (genetic) postmating isolating barriers in the F1 (or F2) hybrids (Diabate et al. 2007; Hahn et al. 2012). Nevertheless, SNP genotyping at ∼400 000 markers across the genome in paired M and S samples from natural populations in West and Central Africa revealed exclusive form-specific clustering, and the largely shared pattern of M-S differentiation across sampling locations suggested that each form is evolving collectively across this large study area regardless of localized M-S gene flow (Neafsey et al. 2010; Reidenbach et al. 2012). This and other evidence led to the recent naming of M and S as distinct species (Coetzee et al. 2013), hereafter referred to as *An. coluzzii* (formerly, M form) and *An. gambiae* (formerly, S form).

The persistence of sympatric and synchronous populations of *An. coluzzii* and *An. gambiae* despite imperfect premating barriers suggests that ecologically based barriers contribute to their isolation. In fact, there is mounting evidence that the ecology of the aquatic larvae plays a significant role. In the more arid savannas of West Africa (e.g. Burkina Faso and Mali) where the bulk of studies have been conducted, differences between *An. coluzzii* and *An. gambiae* in larval behaviours that affect the risk of predation may help explain why *An. coluzzii* is better adapted to live in ecologically more complex and stable habitats, where predators are more abundant and diverse compared to the ephemeral predator-barren larval habitats of *An. gambiae* (Diabate et al. 2005; Gimonneau et al. 2010, 2012a,b). These studies were spurred by the appreciation of landscape-level heterogeneities in the spatial distribution of the two species in West African savannas, where *An. coluzzii* prevails in more xeric areas characterized by larger, more permanent, aquatic habitats, generally of anthropogenic origin and often associated with irrigation (e.g. rice paddies) (Costantini et al. 2009; Gimonneau et al. 2012a).

However, detailed knowledge about the distribution and habitat segregation of *An. coluzzii* and *An. gambiae* remains fragmentary at a continental scale. Outside of Burkina Faso and Mali, only in Cameroon have the ecological factors underlying the divergence of *An. coluzzii* and *An. gambiae* been investigated (Lee et al. 2009a; Simard et al. 2009; Kamdem et al. 2012), and initial results suggest important and unanticipated regional differences. Contrary to the situation in West Africa, only *An. gambiae* occurs in the drier northern savannas of Cameroon (Simard et al. 2009). In southern Cameroon where *An. coluzzii* and *An. gambiae* co-occur, strong habitat segregation exists along gradients of urbanization in the interior of the country, with *An. coluzzii* prevailing in the core of densely urbanized settings in more stable but polluted larval habitats (Kamdem et al. 2012), in accordance with its higher tolerance of ammonia (Tene Fossog et al. 2013). On the other hand, at sites closest to the Cameroon coast, *An. coluzzii* prevails regardless of the degree of urbanization (Simard et al. 2009; Kamdem et al. 2012), consistent with the results of entomological surveys conducted piecemeal in individual African countries bordering the Gulf of Guinea (Awolola et al. 2005; Calzetta et al. 2008; Caputo et al. 2008; Ridl et al. 2008; Vezenegho et al. 2009; de Souza et al. 2010; Djogbenou et al. 2011).

Species distribution models (SDMs) can be used in many aspects of fundamental and applied ecology and evolution (Peterson et al. 1999; Guisan and Zimmerman 2000). When SDMs are applied to study closely related species, these models can be helpful in exploring biogeographic relationships and niche evolution (Peterson et al. 1999; Losos et al. 2003). Comprehensive species distribution modelling of *An. coluzzii* and *An. gambiae* across their shared range in West and Central Africa has not been attempted before now, and there exist large gaps in our understanding of their biogeographic relationships, constraining the ability to target species-specific control to vectors known to differ in their degree of insecticide resistance and susceptibility to *Plasmodium* (Santolamazza et al. 2008; Ndiath et al. 2011; White et al. 2011a; Dabire et al. 2012). In this study, we aimed to (i) build a model capable of predicting predominance of one or the other species across their sympatric range and (ii) identify ecological factors and physiological correlates that may have shaped the habitat segregation of *An. coluzzii* and *An. gambiae*. Using a large set of distribution records extracted across the study area from published and unpublished data, in conjunction with layers of geographic information that included climatic, spatial and anthropogenic variables, we developed an SDM mapping the relative probability of occurrence of the two species across Western and Central Africa. This model predicts significant habitat segregation between *An. coluzzii* and *An. gambiae* at this continental scale, with *An. coluzzii* showing a ‘bimodal’ distribution of relative abundance resulting from its predominance not only in its well-known xeric habitat in West African savannas, but also along the coastal fringe of West and Central Africa. As the variable ‘distance to coast’ was among the significant predictors of the relative abundance of the two species, we compared the salinity tolerance of coastal and inland populations to identify a physiological mechanism potentially underlying the coastal distribution of *An. coluzzii*. Based on assessments of the acute dose–mortality response after exposure to increasing concentrations of salt, we confirmed that *An. coluzzii* larvae were more tolerant than *An. gambiae*, suggesting a continuing role for salinity tolerance in ecological diversification of the *An. gambiae* complex. Moreover, we found that salinity tolerance increased in both species from their baseline level – corresponding to that of rural populations – whenever larvae were collected from aquatic urban habitats, suggesting a role for osmoregulation in local adaptation to anthropogenic pollutants. Finally, by matching degree of salinity tolerance with levels of syntopy, we found evidence of ecological character displacement between this cryptic species pair, indicating the occurrence of competition-driven divergent natural selection in this trait.

## Materials and methods

### Study area and occurrence records

**Figure 1:**
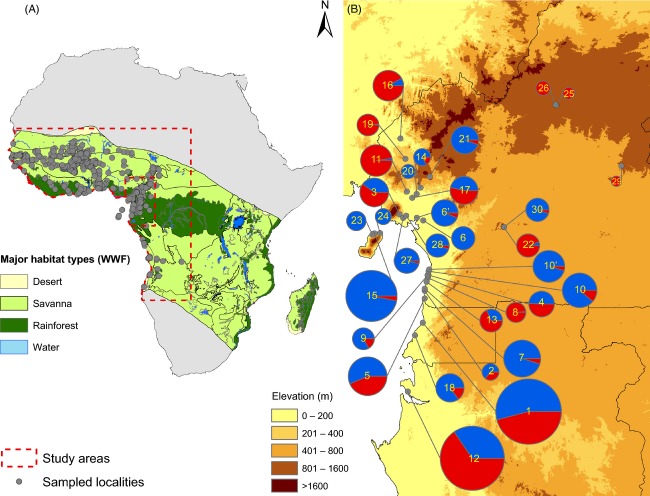
(A) Map of the study area showing major eco-regions (Olson 2001) and the sampled localities included in the data set (dark grey dots). Light grey area falls outside the limits of distribution of *An. gambiae* (Sinka et al. 2010) and was not included in species distribution modelling. The outer dashed line delineates the approximate limits of the area of sympatry of *An. gambiae* and *An. coluzzii*. The inner dashed line delimits the study area in Central Africa where the two species were tested for salinity tolerance. (B) The study area in Central Africa showing localities from which larvae were collected for salinity tolerance testing. The pies show the relative frequency of the two species in larval samples from each locality (blue: *An. coluzzii*; red: *An. gambiae*). The size of the pie is proportional to the size of the sample. Geographic coordinates of localities are given in Table S1. Toponyms referring to the ID number of each locality shown inside the pies are presented in the legend in Fig.2.

*Anopheles gambiae* and *An. coluzzii* are broadly sympatric over much of West and Central Africa (della Torre et al. 2005), across a total surface of ∼8 000 000 km^2^ (Fig.1A). *Anopheles gambiae* occurs allopatrically in East Africa, although there is a single record of *An. coluzzii* occurrence in Zimbabwe (Masendu et al. 2004). In the absence of further such records, we excluded East Africa. Employing an estimate of the eastern boundary of *An. gambiae-An. coluzzii* sympatry (see Fig.1A), we based the analysis on this shared West and Central African range. The study area covers highly diverse habitats, including coastal mangroves, the Guineo-Congolese equatorial rainforest block, forest-savanna mosaic and different types of savanna characterized by increasing degrees of aridity culminating in xeric and predesertic steppes at higher latitudes.

Distribution data for *An. gambiae* and *An. coluzzii* were gathered from various published and unpublished sources reporting about their occurrence and abundance. The data set consisted of 1140 georeferenced localities across the study area sampled during 2001–2011 and included a total of 45 834 *An. gambiae sensu lato* (i.e. *An. gambiae* and *An. coluzzii*) that were molecularly identified to species using rDNA-based PCR assays (Scott et al. 1993; Fanello et al. 2002; Santolamazza et al. 2004) (Table1, Fig.1A).

**Table 1:** Summary of localities and mosquitoes used to model the geographic distribution in relative probability of occurrence of *An. gambiae* versus *An. coluzzii*

Species	No. of mosquitoes (*N*)	No. of localities (*n*)		
		Allopatric	Sympatric	Total
*An. coluzzii*	24 592	151	518	669
*An. gambiae*	21 242	471	518	989
Grand Total	45 834	622	518	1140

### Environmental predictors

Thirteen eco-geographic variables, classified into three factors, were considered as predictors: spatial (four variables), climate (six variables) and land cover (three variables) (Table2). Spatial variables included geographic coordinates for latitude, longitude and their product, to describe the spatial structure of species occurrence (Legendre and Legendre 1998). Based on regional or country-wide entomological surveys indicating a predominance of *An. coluzzii* at sites nearest the coast in several West and Central African countries (Awolola et al. 2005; Calzetta et al. 2008; Caputo et al. 2008; Ridl et al. 2008; Simard et al. 2009; Vezenegho et al. 2009; de Souza et al. 2010; Djogbenou et al. 2011), we also included the variable ‘distance to coast’ as a predictor.

**Table 2:** Eco-geographic predictors used to model the relative probability of occurrence of *An. gambiae* and *An. coluzzii*

Class	Predictor	Description	Wald test value	Odds ratio	95% CI
Spatial	Lat	Latitude	**20.70**	**1.29**	±**0.11**
	Lon	Longitude			
	Lat × Lon	Product of Latitude and Longitude			
	Coast	Distance to Coast (km)	**8.40**	**1.36**	±**0.60**
Climate	Temp Mean	Annual Mean Temperature (°C)	**27.29**	**0.85**	±**0.06**
	Temp Quarter	Mean Temperature of Wettest Quarter (°C)	**23.97**	**1.12**	±**0.04**
	Precipitation Mean	Mean Annual Precipitation (mm)			
	Precipitation Quarter	Precipitation of Wettest Quarter (mm)			
	Dry Season	No. Months with Rainfall < 150 mm			
	Elevation	Mean Elevation (m)	**26.06**	**0.99**	±<**0.01**
Land cover	NVDI Mean	Annual NVDI	**10.86**	**0.98**	±**0.01**
	NVDI Variation	Annual Variation in NVDI	**5.03**	**1.07**	±**0.06**
	Urban	Night Light Intensity			

Bold font indicates variables retained at *P *< 0.05 in the final minimal adequate model. 95% CI: 95% confidence interval of odds ratio.

Climatic variables were annual estimates of mean temperature, mean temperature of the wettest quarter, mean precipitation, mean precipitation of the wettest quarter and length of the dry season (defined as the number of months when rainfall is below 150 mm). Elevation was included as a proxy for smaller scale climatic heterogeneities. Climatic variables and elevation were obtained from the Worldclim project database [http://www.worldclim.org (Hijmans et al. 2005) at ∼1 × 1 km spatial resolution].

Land cover variables were based on the Normalized Difference Vegetation Index (NDVI). The NDVI captures the abundance and chlorophyll content (as a proxy of plant growth state and vigour) of vegetation on the land surface, parameters which reflect soil moisture. The NDVI has been successfully employed to highlight changes in land cover over space and time (Nicholson et al. 1990; Nicholson and Farrar 1994). We derived the NDVI from remotely sensed imagery (https://lpdaac.usgs.gov/products/modis_products_table/mod13a3; ∼1 ×1 km spatial resolution) collected from June to October (the wet season in the savanna belt of West Africa) over a 13-year period spanning 1998–2010. One image was obtained for each 16-day period; thus, 10 images were analysed per annum. Two different NDVI-derived variables were calculated: the annual NDVI mean of the wet season and interannual variation of the seasonal NDVI mean (quantified as the coefficient of variation of yearly seasonal means). A third land cover variable, the intensity of skylight at night, was used as an index of the degree of urbanization and human impact over the natural landscape. It was extracted from satellite images available at http://ngdc.noaa.gov/eog/others/download_world_change_pair.html (∼1 × 1 km spatial resolution).

In relation to average *An. gambiae* dispersal ability (Costantini et al. 1996), we chose 5 × 5 km cells as the spatial resolution for our analysis. Thus, all environmental predictors were rescaled from their native spatial resolution to 5 × 5 km cells. The new value assigned to a given 5 × 5 km cell was the average of values in the input 1 × 1 km pixels within the cell.

### Species distribution modelling

Statistical models to distinguish between populations of *An. gambiae* and *An. coluzzii* were developed. As a first step, we fitted logistic regressions to a subset of training localities where the two species occurred allopatrically (*n *= 622; Table1) (Hosmer and Lemeshow 2000): for localities where only *An. coluzzii* or *An. gambiae* was recorded, the response variable was set to one or zero, respectively. To identify the subset of significant eco-geographic predictors explaining species habitat segregation, we used a forward–backward stepwise model selection procedure. Progressively more parsimonious models (those with fewer predictors) were selected if they decreased the Akaike information criterion [AIC; (Akaike 1974)], until the minimal adequate model – the one returning the lowest AIC – was identified (Acevedo et al. 2010). Its predictive performance was evaluated using the subset of localities where both species were recorded in sympatry (*n *= 518; Table1) as an independent data set. For this purpose, we used calibration plots (Pearce and Ferrier 2000) in which the observed frequency of *An. coluzzii* (relative to *An. gambiae*) was regressed against the predicted probability of (relative) *An. coluzzii* occurrence (Zheng and Agresti 2000). Given complete coincidence between observations and predictions (i.e. perfect calibration), the regression line is expected to pass through the origin with a slope of one (Miller et al. 1991). The range of predicted probabilities of occurrence [0,1] was partitioned in ten equidistant classes of bins, and the mean predicted probability and corresponding mean observed frequency were calculated within each bin class. In estimating the calibration regression parameters, we excluded those bins where the number of localities was *n *< 15, the minimum sample size necessary to yield mean frequency estimates within ±15% of the true mean (Jovani and Tella 2006).

### Mosquito larval sampling and salinity tolerance tests

Larval salinity tolerance was investigated in thirty-two populations from coastal and inland localities lying in the rainforest eco-climatic domain of southern Cameroon, Equatorial Guinea and Gabon, Central Africa (Fig.1B; Table S1). About 170 water collections representing individual mosquito breeding sites were sampled, yielding ∼15 800 *An. gambiae s.l*. larvae (i.e. a mix of *An. coluzzii* and *An. gambiae* in this study area) that were subsequently tested for salinity tolerance (Table S1).

**Figure 2:**
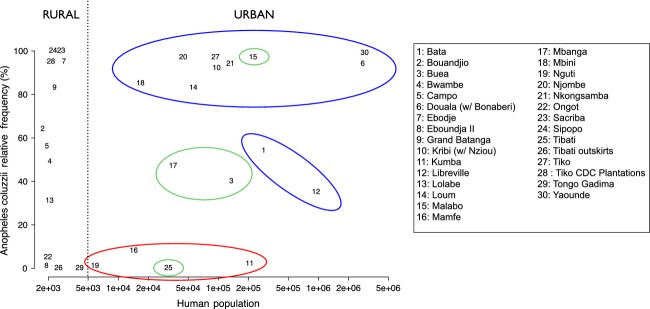
Relationship between the size of each locality shown in Fig.1B, expressed as number of inhabitants and the relative abundance of *An*. *coluzzii* and *An. gambiae*. Ellipses identify urban centres with respect to their position relative to the Cameroon Volcanic line (CVL): westwards (red), eastwards (blue) or on the CVL (green). On the opposite sides of the CVL, urban centres are dominated by one or the other of the two species, while localities on the CVL identify a ‘contact zone’. Exceptions to this pattern are Bata (N°1) and Malabo (N°15) in Equatorial Guinea, Libreville (N°12) in Gabon and Tibati (N°25) in Cameroon. The latter is situated on/east of the CVL, but it lies at >800 m altitude in the forest/savanna mosaic, outside of the rainforest ecozone. In Bata and Libreville, the two species are in apparent parapatry (data not shown). Malabo is on the island of Bioko, on the CVL, under the influence of insular biogeography (e.g. Deitz et al. 2012).

Sampled localities were selected based on two criteria: (i) type of habitat with respect to degree of urbanization (i.e. rural versus urban) and (ii) ecological proximity to saltwater (i.e. the sea or river estuaries), so that both coastal and inland locations were included in the study. Based on previous results (Kamdem et al. 2012), urban locations were defined as those hosting ≥5000 inhabitants and urban infrastructures such as paved roads and sewage collection ditches; inland locations were defined as those lying ≥50 km from the coastline or ≥200 m above sea level. Sampled rural breeding sites included pools, rain puddles, ruts, ditches and stranded pirogues containing residual sea water diluted by freshwater. Urban larval habitats included rain puddles, pools and ditches, often located near dumping grounds; accordingly, many of these breeding sites showed signs of apparent organic pollution (filamentous algae, cyanobacterial mats, pungent odours, decomposing waste, etc.) in the slums of urban areas. Only breeding sites returning a sufficient number of late-instar larvae (cf. below) were selected for the tests. Because of the way these taxa partition their habitat in this eco-geographic region, both species occurred in each locality either in allopatry, sympatry or parapatry according to geography and degree of urbanization (Fig.2); when in sympatry, they often co-occurred in the same breeding sites in more or less balanced proportions. The number of breeding sites tested from each locality varied according to their local abundance (range: 1–27; median: 5; interquartile range: 2.25–7), as sampling effort (i.e. the number of man × hours devoted to larval collections in a single locality) was kept approximately constant across localities. Exceptions were represented by the major urban centres of this region (Bata, Bonaberi/Douala, Libreville, Malabo, Yaoundé – Figs1B and 2), which were also part of a broader scale study on the urban ecology of malaria vectors (Kengne et al., unpublished data); accordingly, these towns were generally represented by a greater number of breeding sites due to their inclusion in multiple surveys (Table S1).

To relate tolerance with the salinity of the natural breeding sites where each species occurred, larval sampling was accompanied by measurements of ions concentration in individual larval habitats using a portable field tester (Wagtech conductivity meter CyberScan CON11 C551-210), which normalizes readings of electrical water conductivity (EWC) at 25°C.

Mosquito larvae were dipped (Service 1993) from individual breeding sites and then brought to the laboratory or field facilities for salinity tolerance testing on the same day of collection. Before assaying, *An. gambiae s.l*. larvae were sorted out from other species of mosquitoes under a dissecting scope according to a morphological identification key (Gillies and Coetzee 1987). *Anopheles coluzzii* and *An. gambiae* larvae are morphologically undistinguishable; so at this point of the experimental protocol, it was not feasible to distinguish which of the larvae were *An. gambiae* and which *An. coluzzii*. Except in cases of known habitat segregation (e.g. Kamdem et al. 2012), each batch of test mosquitoes (usually) contained a mix of both species in variable and *a priori* unknown proportions, and it was only through *a posteriori* DNA-based molecular identification of tested larvae that it was possible to count how many *An. coluzzii* or *An. gambiae* died or survived at each salinity concentration.

Larvae from each individual breeding site were kept and tested separately from those collected from other sites. The pool of larvae collected from a breeding site was randomly allocated to seven cups representing increasing test concentrations of commercial sodium chloride (refined large-grained table salt containing no additives) in distilled water. Six cups contained, respectively, 5%, 10%, 20%, 30%, 40% and 50% dilutions of a 35 g/L NaCl solution assumed to represent 100% sea water. The seventh cup contained only distilled water to act as a control (0% sea water). Occasionally, other concentrations were also tested (35%, 45%, cf. Table S1). Depending on the yield of individual field collections, which varied from one breeding site to another as a function of larval density, the number of larvae tested in each cup ranged from a minimum of 1 to a maximum of 25 (median: 14; interquartile range: 10–17). We defined each test cup as a single batch and the whole series of batches of increasing salinity concentrations from the same individual breeding site as an experimental replicate.

To minimize pupation during the assays, we selected late 3rd to early 4th instar larvae. Before transfer into the test solution, these were fed for 2 h in distilled water with baby-fish food to avoid cannibalism and standardize as much as feasible the physiological status of tested larvae, after which they were not fed. In the field, tests were run indoors at ambient temperature; in Yaoundé, tests were carried out in the laboratory, where temperature averaged 25°C. After 24 h exposure, all larvae that did not react with an escape response (swimming and/or diving) upon stimulation with the tip of a pipette were scored as dead.

### Molecular identification of *An. gambiae sensu lato* larvae

At the end of the exposure interval, all larvae were killed and preserved in microtubes containing 70% ethanol to assess their taxonomic status. DNA of individual larvae extracted by a cetyl trimethyl ammonium bromide (CTAB) protocol (Morlais et al. 2004) was targeted for species identification based on a ribosomal DNA-based PCR-RFLP assay (Fanello et al. 2002).

### Statistical analysis of dose–mortality response to salinity

To assess the dose–response and calculate the median lethal concentration (LC_50_) for different habitats (rural versus urban), locations (coastal versus inland) or species (*An. coluzzii* versus *An. gambiae*), we fitted binomial generalized linear mixed models (GLMM) with a logit link function using the *lme4* package in R v.2.15.3. We fitted ‘salinity concentration’, ‘habitat’, ‘location’ and ‘species’ as fixed effects in the GLMMs; the random effects were ‘salinity concentration’ included as a linear model term grouped by ‘locality’ and ‘breeding site’, with ‘breeding site’ nested within ‘locality’. In this way, a separate logistic regression with random intercept and slope was fitted to each individual breeding site to account for heterogeneities and nonindependence of the dose–response among breeding sites from the same locality, as well as in the average dose–response among different localities. From the intercept and slope of the fixed effects, the median lethal concentration (LC_50_) of the main factors of interest (habitat, location and species) was calculated. In comparing the magnitude of fixed effects, we defined per cent relative difference between two values *x* and *y* as | *x* – *y *| ÷ max(*x*,*y*).

Inference on the size of fixed effects was based on an information-theoretic approach (Burnham and Anderson 2002, 2004): the set of competitive statistical models that were evaluated included those having the random effects and all possible combinations of fixed effects and their second- and third-order interactions conditional to marginality constraints of main effects. After having verified that the fitted models did not show overdispersion, these were sorted according to increasing values of the second-order bias correction Akaike information criterion (AIC_c_), as appropriate for models in which the ratio of number of observations *n* to number of parameters *K* is *n*/*K *≤ 40. The ΔAIC_c_ statistic was calculated as the AIC_c_ difference between a focal model and that having the minimum AIC_c_. The best model has ΔAIC_c_ = 0, but models considered to have substantial statistical support were identified by the rule of thumb ΔAIC_c_ ≤ 2 and the analysis of Akaike weights (Burnham and Anderson 2002, 2004). Whenever there was evidence for more than a single best model, we calculated multimodel average parameters. We used full-model averages with shrinkage, which do not have a tendency to bias the value away from zero. As it is an unresolved issue how the variance of these estimates should be calculated, the standard errors and confidence intervals were returned for the subset-averaged (or ‘conditional’) coefficients. All calculations were carried out using functions available in the R package *MuMIn* v.1.9.0.

## Results

### Species distribution models

The eco-geographic predictors retained in the final minimal adequate model are summarized in Table2. The stepwise procedure selected 7 of 13 variables representative of all three factors (i.e. spatial, climate and land cover), which explained 64% of the overall variance in the data (estimated from the Nagelkerke coefficient of determination *R*^2^).

**Figure 3:**
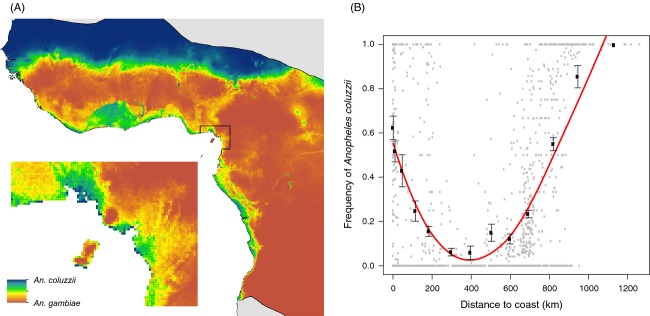
(A) Relative probability of occurrence predicted for *An*. *gambiae* and *An. coluzzii* (dark blue: 1 *An. coluzzii*, and 0 *An. gambiae*; dark red: 1 *An. gambiae*, and 0 *An. coluzzii*) across their sympatric range. The inset shows an enlarged view of the area delimited by a black line in the map where high altitudes are encountered close to the coastline due to the presence of Mount Cameroon and the Cameroon Volcanic line; (B) Frequency of *An*. *coluzzii* relative to *An. gambiae* plotted as a function of distance from the coast. The red line depicts the second-order polynomial regression curve fitted to the observation records, represented by grey dots. Black circles and associated standard error bars illustrate the mean frequency of *An*. *coluzzii* for arbitrary classes of distance plotted for a visual assessment of goodness-of-fit.

The minimal adequate model was projected across the study area, producing a map of predicted relative probability of occurrence of *An. coluzzii* relative to *An. gambiae* (Fig.3A). One of the main features of this map is the ‘bimodal’ distribution of *An. coluzzii* in contrasting geographic domains: the highest probabilities of occurrence correspond to a wide belt covering the most xeric savannas found at higher latitudes in Western Africa – from the westernmost limit in Senegambia and eastwards to Chad – and along a narrow strip of coastline bordering the Gulf of Guinea, regardless of the precise environmental domain encountered along this strip. If it were not for a zone of contact between these two regions, corresponding to the coastal savanna areas at the westernmost limit in Senegambia, the range of *An. coluzzii* would approximate a disjunct distribution, particularly at its easternmost limits.

To provide better visual and quantitative assessment of the ‘bimodality’ in probability of occurrence of *An. coluzzii*, we plotted its relative frequency across all localities (i.e. training + validation data sets) as a function of distance from the coast (Fig.3B). The highest relative frequencies occurred at the shortest and longest distances. In the coastal zone within 5 km of the sea, the average observed relative frequency of *An. coluzzii* was 0.62 ± 0.05, and beyond 900 km from the Gulf of Guinea the observed relative frequency was 0.88 ± 0.04. The minimum average frequency (0.06 ± 0.04) was found at an intermediate distance, 350–450 km from the coast.

Conversely, *An. gambiae* had the highest relative probability of occurrence across the central block of moist savannas, in the forest/savanna mosaic, in most of the rainforest area and in highlands. Although the dry savannas of West Africa share climatic similarities with the southern arid regions of Angola and Democratic Republic of Congo (DRC), *An. gambiae* had contrasting relative probabilities of occurrence in these two regions – low in western, but high in southern arid savannas. Along major lowland rivers such as the Niger and the Benoue/Donga in Nigeria, the Ogoue in Gabon and the Congo in the DRC, both species have similar (intermediate) relative probabilities of occurrence.

Calibration plots assessing the predictive performance of the final model showed that predictions closely fitted observations (coefficient of determination of the regression line: *R*^2^ = 0.99; Figure S1). There was only a slight amount of calibration bias and spread, indicating that at locations where the predicted probability of occurrence was >∼60% the model slightly overestimated the frequency of *An. coluzzii*, and conversely*,* at localities below the 60% threshold, the model underestimated the relative frequency of *An. coluzzii*.

### Population patterns in salinity tolerance

During the salinity tolerance assays, occasionally we observed unusual mortality rates in the control batches. Thus, we investigated first how larval control mortality was partitioned across habitats, locations and populations by fitting binomial GLMMs only to the control batches. Even if average control mortalities were well below 5% (the arbitrary consensus threshold accepted to validate an acute toxicity bioassay), habitat and location of the test population had an impact on control mortality rates (Table3). The relative importance of the fixed effects was ‘habitat’ = 0.66, ‘location’ = 0.46, and ‘habitat × location’ = 0.14. The full set of models was not well resolved (Table3); hence, we calculated multimodel parameter estimates to fit average control mortalities. Mortality rankings were rural inland < rural coastal < urban coastal < urban inland (Table4). Urban control mortalities were more than threefold higher compared to rural localities, regardless of the location of the population (Table4), potentially reflecting greater levels of stress due to increased exposure to xenobiotics resulting from anthropogenic pollution. To reduce possible biases in subsequent analyses, we excluded all replicates in which the control batch scored at least one death at the end of the 24-h exposure interval. Results from the statistical analysis of the full data set, that is without excluding replicates with mortality in the controls, were qualitatively similar to those presented below (data not shown).

**Table 3:** Evaluation of competitive binomial generalized linear mixed models fitted to control batches of the salinity tolerance assays data set

Model No. (rank)	Model terms (fixed effects)				d.f.	ΔAIC_c_	Akaike weight
	Intercept	Habitat	Location	Habitat × Location			
2 (1)	−6.473	+			4	0	0.349
1 (2)	−4.985				3	1.19	0.193
4 (3)	−6.641	+	+		5	1.35	0.178
3 (4)	−5.431		+		4	1.78	0.143
8 (5)	−5.949	+	+	+	6	1.87	0.137

Fixed effects included in the full model (Model No. 8) are the factors ‘habitat’ (two levels: rural versus urban), ‘location’ (two levels: coastal versus inland) and their interaction. Crosses (+) indicate the occurrence of a term in the model, while the intercept (logit scale) refers to the fitted value for baseline factor levels. Random effects of all models are breeding sites nested within localities. Models are sorted according to increasing values of the second-order bias correction Akaike information criterion (AIC_c_). The ΔAIC_c_ is the AIC_c_ difference between a model and that having the minimum AIC_c_, so that the best model has ΔAIC_c _= 0. Models whose ΔAIC_c _≤ 2 are considered to have substantial statistical support and those whose ΔAIC_c_ > 2 have less to no support. Akaike weights quantify the ‘strength of evidence’ for a given model, that is the probability that the model is the best one, conditional on both the data and full set of models considered.

**Table 4:** Ratio (above the main diagonal) and per cent relative difference (below the main diagonal) of multimodel control mortality estimates observed during the salinity tolerance assays

	Rural		Urban	
	Coastal	Inland	Coastal	Inland
Mean mortality	0.25%	0.05%	0.74%	1.12%
Rural				
Coastal	–	5.4	3.0	4.5
Inland	81%	–	16.0	24.4
Urban				
Coastal	66%	94%	–	1.5
Inland	78%	96%	34%	–

After having verified with the full data set that ‘habitat’ (urban/rural), ‘location’ (inland/coastal), ‘salinity concentration’ and their second- and third-order interactions had significant impacts on larval mortality (Appendix S1), next we fitted binomial GLMMs to test whether salinity tolerance differed between *An. coluzzii* and *An. gambiae*. To do so, we had to take into account two constraints: (i) that results from different populations could not be pooled in a single analysis, as just mentioned above (Appendix S1); and (ii) that molecular identifications were carried out either at batch level for sympatric populations or at breeding site level for all other populations. In the first instance, specific dose–response curves could be estimated, whereas in the latter case, specific parameter estimates were obtained by carefully choosing the populations or breeding sites to be included in the analysis.

Overall, *An. coluzzii* was significantly (Table5) more tolerant than *An. gambiae* to salinity, except when populations of the latter occurred – in fewer instances – in urban environments (Table6). The level of tolerance within species, however, was not predicted by distance of the population from the coast or altitude, as the effect due to urbanization swamped that due to location (Table6; Appendix S1).

**Table 5:** Evaluation of competitive generalized linear mixed models fitted to the salinity tolerance assays data set pertaining to rural coastal populations of *An. coluzzii* and *An. gambiae* from Cameroon and Equatorial Guinea

Model No. (rank)	Model terms (fixed effects)				d.f.	ΔAIC_c_	Akaike weight
	Intercept	Salinity	Species	Salinity × Species			
4 (1)	−11.280	0.3617	+		9	0	0.748
8 (2)	−11.040	0.3538	+	+	10	2.24	0.244
2 (3)	−10.080	0.3442			8	9.12	0.008
1 (4)	1.179				7	25.26	0
3 (5)	−3.762		+		8	42.04	0

Parameter estimates of the model with best statistical support (No. 4) are shown in Table6. For an explanation of statistics and symbols, cf. Table3.

**Table 6:** Dose–response parameters of salinity tolerance for several populations of *Anopheles coluzzii* and *An. gambia*e collected from Cameroon, Gabon and Equatorial Guinea (Central Africa)

Species	Habitat	Location	Intercept (±SE)	Slope (±SE)	LC_50_ (±SD)	Populations*
*An. gambiae*	Rural	Coastal	−9.103 (±0.706#)	0.362 (±0.046)	25.2 (±2.8)	Bouandjo, Bwambe, Ebodje, Eboundja II, Grand Batanga, Lolabe
		Inland	−8.259 (±0.952)	0.350 (±0.053)	23.6 (±2.9)	Ongot, Tibati outskirts, Tongo-Gadima
	Urban	Coastal	−8.587 (±1.176)	0.279 (±0.035)	30.8 (±3.6)	(Bata), Campo, Libreville
		Inland	−8.374 (±1.078)	0.235 (±0.029)	35.6 (±4.2)	Buea, Kumba, Mamfe, Mbanga, Nguti, Tibati
*An. coluzzii*	Rural	Coastal	−11.280 (±1.826)	0.362 (±0.046)	31.2 (±2.8)	Bouandjo, Bwambe, CDC Plantations, Ebodje, Eboundja II, Grand Batanga, Lolabe, Sacriba, Sipopo
		Inland	–	–	–	–
	Urban	Coastal	−8.271 (±0.798)	0.274 (±0.021)	30.1 (±3.6)	Bata, Bonaberi, Campo, Douala, Kribi, Libreville, Malabo, Mbini, Nziou, Tiko
		Inland	−8.833 (±0.673)	0.246 (±0.024)	35.9 (±4.1)	(Buea), Loum, Mbanga, Njombe, Nkongsamba, Yaoundé

The median lethal concentration (LC_50_), intercept and slope refer to the logistic regression lines relating larval mortality to water salinity, expressed in per cent sea water. Dashes indicate lack of data due to the absence of populations from certain habitats (Kamdem et al. 2012). The ‘populations’ column shows the localities that were included in the analysis to calculate specific parameter estimates. When the same locality appears in multiple rows, specific parameters were estimated by taking into account results of molecular identifications at the breeding site level.

* cf. Figures1B, 2 and Table S1; populations in parentheses are not included in the analysis for lack of adequate samples for that specific population.

# SE of the difference of the corresponding parameter estimate for *An. coluzzii* in the same Habitat/Location.

### Salinity tolerance and ionic environment

**Figure 4:**
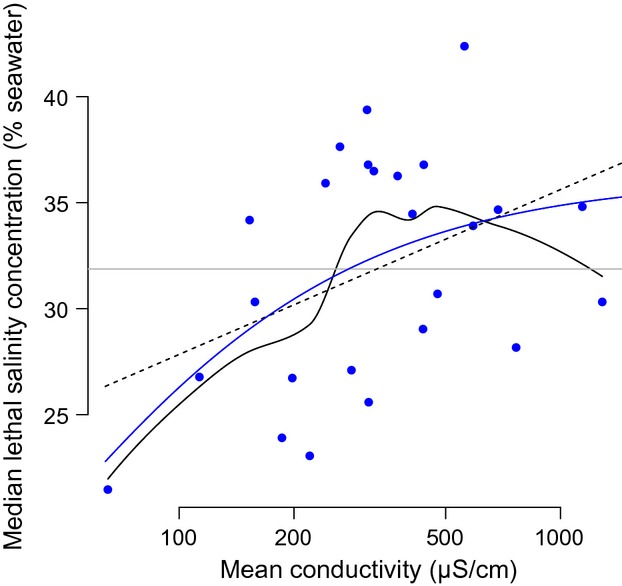
Functional relationship between electrical water conductivity and salinity tolerance in individual populations of *An. coluzzii* and *An. gambiae* from Central Africa. Trend lines show competitive regression models fitted to the data, whose parameters are presented in Table7. The solid continuous black curve represents a loess smoother fitted to the data in order to extract the general shape of the functional relationship between the two variates. The blue asymptotic curve is the most parsimonious model based on the AIC. The other competitive models are shown as a dashed black (linear model) and horizontal grey (null model) lines.

Salinity tolerance may increase in urban habitats (regardless of inland or coastal location) due to higher content of ions derived from pollution by nitrogenous and phosphorous compounds (Antonio-Nkondjio et al. 2011; Tene Fossog et al. 2013). To investigate the relationship between the degree of salinity tolerance and the amount of ions available in the aquatic milieu of natural breeding sites, we plotted the LC_50_ of individual populations as a function of the average electrical water conductivity (EWC) measured in those breeding sites from which test larvae were collected in each locality (Fig.4). The general shape of the functional relationship between the two variates was explored by fitting a generalized additive model to the data. The resulting loess smoother (continuous solid black curve in Fig.4) suggests that salinity tolerance of populations increased with the average conductivity of the breeding sites, and that the relationship plateaus, or perhaps humps down, at the highest EWC values that we measured in the field. To test the significance of this association, as well as whether the relationship is linear or asymptotic, we tested three competitive linear and nonlinear models (Table7). Based on the AIC, the null model (of no association) was rejected, and the linear model had much weaker statistical support compared to the nonlinear model of a positive asymptotic relationship between average salinity tolerance and EWC, with an asymptote estimated at 36.2% sea water (Table7 and Fig.4).

**Table 7:** Parameter estimates of the competitive linear and nonlinear models shown in Figure4

Model name (rank)	Parameter estimates				Residual d.f.	ΔAIC
	Model Equation	*a* (±SE)	*b* (±SE)	Asymptote		
Asymptotic (1)	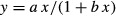	0.9621* (±0.4117)	0.0266* (±0.0127)	36.2	23	0
Linear (2)	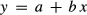	12.29 (±8.80)	7.78* (±3.47)	–	23	2.49
Null (3)	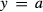	31.9*** (±1.1)	–	31.9	24	5.42

Asterisks denote levels of statistical significance for the null hypothesis that the parameter is equal to zero:

* *P *< 0.05

*** *P *< 0.001.

Notably, mean conductivity values of the sampled larval habitats were higher in coastal compared to inland, as well as in urban compared to rural sites within each location (Figure S2). The difference is close to statistical significance for the comparison between locations (anova, *F*_1,157_ = 3.23; *P *= 0.07). However, the EWC data are distributed log-normally, and more revealing is the variability in EWC among larval habitats within each environment. Importantly, coastal breeding sites were significantly more variable in EWC than inland ones (Bartlett test of homogeneity of variances: *K*^2^ = 68.04; d.f. = 1; *P* < 0.0001), regardless of type of habitat (Figure S2).

## Discussion

Phenotypic divergence can result from divergent or disruptive natural selection arising from differential resource exploitation, competitive interactions and/or ecological opportunity. These processes can be important causal drivers of divergence ultimately leading to speciation (Schluter 2000; Coyne and Orr 2004; Dieckmann et al. 2004). Traits evolving under the influence of divergent natural selection can provide fitness advantages under specific environmental conditions, leading to local adaptation of subpopulations. Adaptation to alternative environments therefore plays a crucial role in generating phenotypic and population divergence and provides a mechanistic ecological understanding of the process of speciation (Orr and Smith 1998; Turelli et al. 2001; Bolnick and Fitzpatrick 2007).

Recently diverged species, for which clues about past speciation history have not yet been completely erased, provide opportunities to test these ideas. The cryptic species pair *An. coluzzii* and *An. gambiae* investigated here represents a good model because their divergence is recent and reproductive isolation has been accomplished to variable degrees across their geographic range (e.g. Nwakanma et al. 2013). As their speciation history cannot be studied directly, it is crucial to understand which traits may be exposed to divergent natural selection and which ecological conditions promote diversification of these traits. Towards this aim, we sought major ecological gradients of divergence between *An. coluzzii* and *An. gambiae;* we did this by looking at habitat segregation at a continental scale using species distribution modelling and then tested for a physiological trait that may underlie such habitat divergence.

### Continental distribution modelling of *An. coluzzii* and *An. gambiae*

Previous studies have mapped the distribution of members of the *An. gambiae* complex on a continental scale, using a variety of approaches (Lindsay et al. 1998; Coetzee et al. 2000; Bayoh et al. 2001; Levine et al. 2004; della Torre et al. 2005; Moffett et al. 2007; Sinka et al. 2010). However, for largely historical reasons, all except one (della Torre et al. 2005) conflated *An. gambiae* and *An. coluzzii* as a single taxon, and Bayoh et al. (Bayoh et al. 2001) focused on chromosomal karyotypes shared to varying extents between the two species (della Torre et al. 2005; Costantini et al. 2009; Simard et al. 2009). The sole study to consider *An. gambiae* and *An. coluzzii* separately collated observation records to provide a qualitative assessment of the distribution of the two species (della Torre et al. 2005). Here, we related known occurrences of these species to environmental variation across landscapes, to develop quantitative models of their relative distribution that allow statistically robust predictions even in unsampled areas (Peterson 2006).

The major global pattern that emerged from this study is the ‘bimodality’ of the predicted relative probability of occurrence of *An. coluzzii* with respect to *An. gambiae*. Its predicted predominance along a broad belt of xeric landscapes at higher latitudes is not surprising, as the association of *An. coluzzii* with aridity has been noted previously, at least in West Africa (della Torre et al. 2005; Costantini et al. 2009), and is consistent with its superior resistance to desiccation stress relative to *An. gambiae* (Lee et al. 2009b). The fact that climatic variables most obviously related to degree of aridity (those concerning amount of precipitation) were not significant predictors in our model does not necessarily result from a lack of influence of these variables on the fundamental ecological niche and physiological limits of the two species. Rather, it seems more likely that degree of aridity was captured by the spatial predictor ‘latitude’, the climatic predictor ‘temperature’ (two variables: ‘temp mean’ and ‘temp quarter’) and the land cover predictor ‘NDVI’ (two variables: ‘NDVI mean’ and ‘NDVI variation’) and that precipitation-related climatic variables did not provide additional significant explanatory power beyond that afforded by latitude, temperature and NDVI once the latter were fitted into the model.

The unexpected and conspicuous second ‘mode’ of the predicted bimodal predominance of *An. coluzzii* is a ribbon of coastline bordering the Gulf of Guinea, largely captured by the spatial descriptor, ‘distance to coast’. This is visualized in the map of relative probability of occurrence (Fig.3A) as a thin strip of *An. coluzzii* predominance along the whole coastline of West and Central Africa, expanding inland to a considerable extent in the forested region between Côte d'Ivoire and Ghana potentially due to the influence of altitude (see below). The steep drop in *An. coluzzii* relative frequency when moving away from the coastline is especially striking in light of the fact that the localities serving to fit the parabolic relationship between relative frequency and distance from the coast (Fig.3B) do not fall along a linear transect, but instead are spread across a vast geographic area. This consideration underscores the homogeneity of the coastal distribution pattern, which may reflect the existence of a coastal population of *An. coluzzii* that is isolated to some extent from northern savanna populations.

The global pattern of relative probability of occurrence of *An. coluzzii* along the coastline appears to be modulated locally by environmental heterogeneities such as altitudinal gradients, along which *An. coluzzii* and *An. gambiae* segregate. This can be seen clearly considering the arc of ancient volcanoes emerging as oceanic islands in front of Mt. Cameroon (including Bioko Island) and present-day volcano Mt. Cameroon itself, whose slopes emerge from the coastline and rise to more than 4000 m above sea level. The data predict a reversal in the relative abundance of the two species along such altitudinal gradients near the coast: *An. coluzzii* predominates at sea level, but is replaced by *An. gambiae* above ∼430 m (Fig.3A). This predicted pattern conforms to the observed relative abundance of *An. coluzzii* and *An. gambiae* in the region of Mt. Cameroon and the backbone of the Cameroon Volcanic Line. *An. coluzzii* is predominant, as expected, in localities such as Tiko, Limbe or Idenau occurring along the coast at elevations <200 m (Bigoga et al. 2007; Lee et al. 2009a; Simard et al. 2009); conversely, its abundance decreases below the threshold of detection in mosquito samples above 800–1000 m (Tanga et al. 2010). At intermediate elevations, such as Buea, both species can be present in balanced proportions (Tanga et al. 2010; this study, Figs1B, 2 and 3A). The association of coastal *An. coluzzii* populations with low altitude may explain the expanded patch of predicted suitable habitat between Ghana and Côte d'Ivoire (Fig.3A), an area mostly lying below 200 m elevation. Conversely, the absence of *An. coluzzii* beyond 800–1000 m elevation may explain its surprising absence in xeric savannas at higher latitudes in the southern hemisphere (e.g. in central and south-eastern Angola), which are mainly highland plateau regions above 800 m elevation. At these higher altitudes, patches of *An. coluzzii* apparently occur only in strictly urban environments (e.g. Yaoundé and Nkongsamba at 800–850 m in Cameroon or Huambo at 1700 m in Angola [Cuamba et al. 2006; Calzetta et al. 2008; Kamdem et al., 2012; this study]).

Somewhat surprisingly, the major environmental predictor ‘degree of urbanization’, which was identified by species distribution models developed from regional and micro-geographic surveys conducted in central and southern Cameroon (Kamdem et al. 2012), was not a significant predictor in our continental-scale SDM. This apparent contradiction may be explained by several factors, both technical and biological. First, the geographic scale of analysis differs [1 × 1 km in Kamdem et al. (2012) vs 5 × 5 km spatial units in this study]. Given that reversals in the relative abundance of the two species can happen along urban–rural transects as narrow as 6 km (Kamdem et al. 2012), the spatial resolution of the present study may have blurred the effect of urbanization on probability of occurrence. Second, the impact of urbanization on *An. coluzzii* prevalence has been extensively studied and validated only in populations occurring in the rainforest eco-climatic domain in Cameroon, Central Africa. The impact of this environmental variable may be smaller (or nil) in the xeric savannas in Western Africa. In fact, it is noteworthy that our samples from larval populations located in urban habitats west of the Cameroon Volcanic line were predominantly *An. gambiae*, not *An. coluzzii* (Figs1B and 2). The Cameroon Volcanic line is a geographic barrier structuring populations of *An. coluzzii* (Pinto et al. 2013) and at least one other species of the *An. gambiae* complex, *An. melas* (Deitz et al. 2012). Third, as most of the largest cities of the rainforest ecozone in Western and Central Africa are coastal, the degree of urbanization is statistically confounded with the other spatial predictor used in our SDM, ‘distance to coast’.

### The ecological significance of differences in salinity tolerance

In addition to comprehensive modelling of *An. coluzzii* versus *An. gambiae* distributions, a second major objective of this study was to identify physiological correlates that may have shaped the predicted distribution patterns. In particular, we aimed to test the hypothesis that *An. coluzzii* has greater tolerance to salinity than *An. gambiae*, a capacity that may contribute to its predicted coastal distribution along the entire Gulf of Guinea and its observed coastal distribution in southern Cameroon. Acute toxicity bioassays demonstrated moderate but statistically significant differences in larval salinity tolerance between the two species whenever they co-occurred in strict sympatry in rural habitats, as well as between rural and urban populations independent of distance to the coast. Both coastal and inland populations of *An. coluzzii* expressed higher tolerance to salinity than *An. gambiae*, except in instances when the latter species occurred in urban habitats. Although beyond the scope of the present study, further insights could be gained from investigations of chronic exposure to salinity and its impact on life history traits and fitness of natural populations.

Relative to the *bona fide* saltwater tolerant sibling species *An. melas* and *An. merus* that develop successfully in brackish water (Gillies and De Meillon 1968; Mosha and Mutero 1982), *An. coluzzii* even at its most salt-tolerant manifestation must be considered an obligate freshwater taxon, confined to water whose osmotic concentration is less than larval haemolymph (∼300 mOsm/kg or ∼30% sea water; Bradley 1987). Nevertheless, increased salinity tolerance in *An. coluzzii* may be ecologically relevant, as it may provide a mechanism to breed more successfully in marginal freshwater habitats prone to contamination by salts, thereby conferring an advantage against less tolerant competitors such as *An. gambiae*. For example, larval habitats exclusive to *An. coluzzii* during our surveys were fishermen pirogues stranded on the beach or emerging water table pools along the beach or river estuaries, which contained much greater amounts of salts. Given the predicted coastal distribution of *An. coluzzii* along the vast Gulf of Guinea, we hypothesize that higher salt tolerance may be a more global attribute across coastal *An. coluzzii* populations, and if so, that this difference may play a role in hitherto unexplained features of the geographic distribution of *An. coluzzii* and *An. gambiae* elsewhere in Central and West Africa. For example, *An. coluzzii* and *An. gambiae* have been reported to segregate along the river Gambia according to eco-geographic gradients (Caputo et al. 2008, 2014): the frequency of *An. coluzzii* decreased when moving away from the river banks and upstream, in accordance with a gradient in the degree of exposure to tidal brackish water. These same studies highlighted the association of *An. coluzzii* with coastal rice cultivation, where this species would be exposed to brackish water. In another example from coastal Benin, the saltwater tolerant species *An. melas* was found to co-occur in breeding sites of relatively high salinity together with a taxon that was almost certainly *An. coluzzii* (unknown at the time; Akogbeto 1995; Djogbenou et al. 2011). Together, these biogeographic records suggest that the greater degree of salinity tolerance measured in *An. coluzzii* from Central Africa may be more widely applicable geographically and combined with other evidence, prompt a more general working hypothesis: *An. coluzzii* is adapted to tolerate greater levels of environmental stressors in its larval habitats, not only in sites closely associated with the coast/sea, but also those associated with urban centres containing xenobiotics and ammonia (Kamdem et al. 2012; Tene Fossog et al. 2013; Cassone et al. 2014). Extending bioassays of *An. coluzzii* and *An. gambiae* across their predicted geographic range will be a critical next step towards confirming this inference.

**Figure 5:**
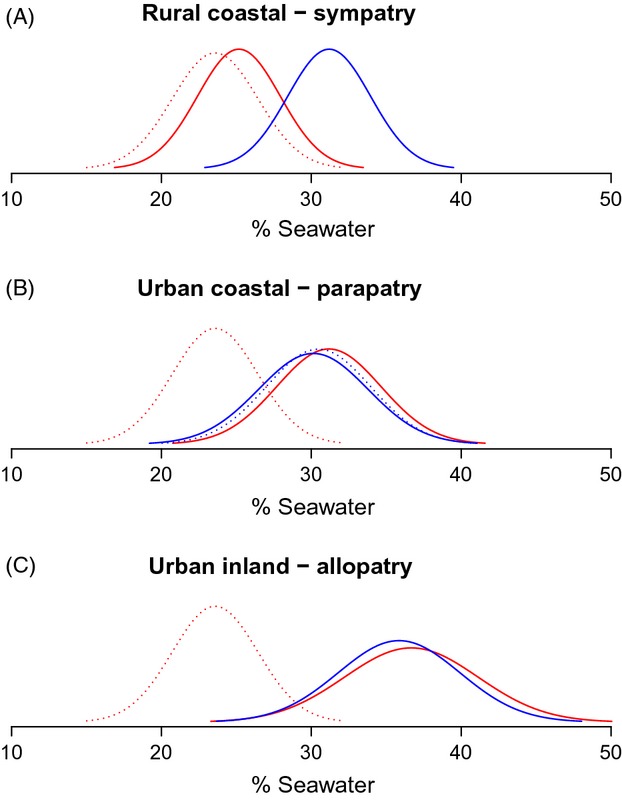
Evidence for ecological character displacement in populations of *An. gambiae* (red) and *An. coluzzii* (blue) from Central Africa. The Gaussian curves show the probability density functions of tolerance thresholds, according to habitat, location and degree of syntopy. The dotted red curve in all panels depicts the tolerance of inland rural forest populations of *An. gambiae*, which are postulated to represent the ancestral state of the trait. Panel (A) includes localities where both species extensively share the same breeding sites, whereas in (B), both species share the same locality but not the same breeding sites due to spatial segregation (Libreville, Gabon). The dotted blue line refers to coastal urban populations of *An. coluzzii* in allopatry, where *An. gambiae* does not occur (Bonaberi/Douala, Malabo, Tiko). In (C), both species share the same habitat but not the same localities, each locality being exclusive of one of the two taxa; *An. gambiae* urban populations are represented by towns located in the savanna (Tibati, Cameroon) or in the forest westwards of the Cameroon Volcanic line (cf. Figs1–1 and Table S1).

Rather paradoxically, urban coastal populations were consistently less tolerant than urban inland ones in both species. Only in the case of *An. gambiae* did rural populations conform to the expectation that inland populations would be less saline-tolerant than coastal ones. Moreover, *An. gambiae* exhibited a twofold more variable degree of tolerance than* An. coluzzii* across populations (Coefficient of Variation: 19.0% versus 9.5%, respectively). To shed more light on these results, it is informative to present salinity tolerance in the context of joint species distributions (Fig.5). Considering only *An. coluzzii*, its level of salinity tolerance was similar across biotopes. However, the tolerance of *An. gambiae* varied substantially as a function of biotope and degree of sympatry with *An. coluzzii*. In particular, sympatric populations of the two species showed divergent levels of salinity tolerance (with *An. coluzzii* having the greater tolerance), but allopatric or parapatric populations of the two species in urban habitats had similar levels of tolerance, largely due to increased salinity tolerance by* An. gambiae* – one of the signatures of ecological character displacement.

### Character displacement in salinity tolerance

Ecological character displacement occurs when competition imposes divergent selection on interacting species, causing divergence in traits associated with resource use (Martin and Pfennig 2011). This process can facilitate species coexistence, enhance phenotypic differences between sympatric species and promote or even initiate speciation and adaptive radiation (Rice and Pfennig 2010; Martin and Pfennig 2011).

The classic pattern to recognize the existence of character displacement is to observe the degree of phenotypic divergence in allopatric and sympatric populations of the same species pair: when sympatric trait values are more extreme than values occurring in allopatry, there is evidence for character displacement (Brown and Wilson 1956). However, this pattern can emerge also as a consequence of other processes. Schluter and McPhail (1992) have summarized the conditions under which character displacement evolves as an adaptive response to interspecific competition. Because of the involvement several different fields of study, it is generally difficult to test all these conditions in a single analysis, which explains why it has been historically difficult to prove the existence of character displacement (Schluter and McPhail 1993; Losos 2000). Clearly, here we have not tested the full range of conditions; therefore, further work is needed to cover the whole set. Most notably, we do not know whether differences in salinity tolerance have emerged *in situ* under coexistence of the two species, or rather as a result of differences evolved in allopatry followed by subsequent range expansion. In other words, it is difficult with our current data to assess the role played by the observed differences in salinity tolerance in the speciation history of *An. coluzzii* and *An. gambiae*. Experimental manipulations can provide a more stringent test of character displacement (e.g. Tyerman et al. 2008): we expect that if allopatric populations of *An. coluzzii* and *An. gambiae* are placed together, they should compete more strongly than sympatric populations.

### Linking distribution modelling with functional ecology: a theoretical framework for testing *An. coluzzii* versus *An. gambiae* interactions in the forest ecozone of Central Africa

**Figure 6:**
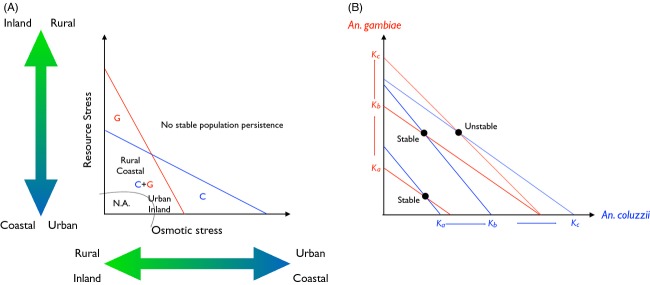
A theoretical framework of functional ecological interactions between *An. gambiae* and *An. coluzzii* in the forest ecozone of Central Africa. Panel (A) represents the zero isoclines of population growth in relation to different environmental stressors: blue (*An. coluzzii*) and red (*An. gambiae*) lines set the limits for combinations of osmotic and resource stress beyond which population growth becomes negative, leading to no stable persistence of either species. On the abscissa, osmotic stress is assumed to increase along the inland versus coastal, as well as the rural versus urban environmental gradients. On the ordinate, stress from lack of resources – particularly nutrients and suitable larval habitats – is supposed to increase in the reverse order along the same environmental gradients. The ‘N.A.’ region delimited in state space by a dotted curve is presumably not available in the study area (i.e. regions with abundant resources in presence of very low levels of osmotic stress). The ‘C’ and ‘G’ letters in state space define regions where *An. coluzzii* and *An. gambiae*, respectively, occur alone, whereas ‘C+G’ represents the region where both species can coexist. However, rural coastal areas have less resources and more variable osmotic stress than urban inland areas, accounting for their different location in state space and differences in carrying capacity *K*_*i*_ shown in panel (B). Panel (B) depicts graphically the outcome of competition generated by the classic Lotka–Volterra equations applied to the *An. coluzzii* versus *An. gambiae* species pair in the ‘C+G’ region of state space in panel (A). The axes refer to the population size of either *An. coluzzii* (blue) or *An. gambiae* (red). As carrying capacities increase moving from small rural to larger and larger urban habitats (*K*_*a*_ < *K*_*b*_ < *K*_*c*_, direction of change indicated by arrows), the equilibrium point of joint population size changes from stable coexistence (cases *a*, *b*, continuous lines) to competitive exclusion (case *c*, dotted lines). For further discussion, see text.

The evidence that salinity tolerance changes in relation to the joint occurrence of *An. coluzzii* and *An. gambiae* in this geographic region prompts a hypothetical working model of species interactions, whose aim is to provide testable working hypotheses for future quantitative research (Fig.6). We assume that larvae of *An. gambiae* and *An. coluzzii* respond differently to two environmental stressors, resource and osmotic stress, such that intersecting isoclines define four regions of population persistence in stressor state space (Fig.6A). For reasons presented above, osmotic stress is assumed to increase in urban and coastal environments. Resource stress corresponds to the breeding opportunities and quality of larval habitats that each biotope provides. In the rainforest of Central Africa, the presence of *An. gambiae s.l*. is strictly dependent on the presence of human activities that clear the forest canopy and expose bare soil for larval habitats to occur; pristine rainforest is not a suitable habitat for the larvae of these mosquitoes. Thus, size of human settlements is taken as a proxy for available breeding opportunities, whereby small rural settlements are generally poorer than urban centres in breeding opportunities, and organic enrichment of larval habitats in denser settlements may increase the bioavailability of nutrients. Similarly, nutrients such as dissolved phosphate, nitrate and silicate can accumulate at coastal river mouths and alluvial plains through drainage and river transport and can lead to eutrophication of surface waters (e.g. Turner & Rabalais, 1994). Thus, we assume resource stress to increase along the urban < rural and coastal < inland environmental gradients (Fig.6A).

Beyond the zero isoclines, population growth is negative, and stable population persistence is not possible except as sink populations. Region ‘C’ in Fig.6A defines the set of values in which osmotic stress is too high for *An. gambiae* despite the abundance and quality of resources (i.e. lower resource stress); this is where *An. coluzzii* occurs alone. Coastal urban habitats are presumed to be mostly representative of this condition. Region ‘G’ defines areas where larval habitats are osmotically favourable but resources too few and poor for *An. coluzzii* to persist stably in the presence of *An. gambiae*; this region in state space is postulated to correspond to inland rural areas in the forest where *An. gambiae* occurs alone. Conversely, in the ‘C+G’ region, both environmental stressors are within the limits of positive population growth for both species; here, *An. coluzzii* and *An. gambiae* can compete for the same resources. In this region of state space, density dependence is likely to modulate the outcome of interspecific competition.

Because of little empirical knowledge, we consider here just the simple case of density-dependent competition modelled by the classic Lotka–Volterra equations (Fig.6B). In rural coastal areas, where populations are smaller due to the lower carrying capacity of this biotope, and the environment is coarse-grained (*sensu* Rosenzweig, 1981) due to a broader spectrum of alternative – marginal – larval habitats (Figure S2), both species can occur in sympatry (stable equilibrium point at *K*_*a*_). Larval habitat selection and character displacement in salinity tolerance contribute to the stable persistence of both species because they reduce interspecific competition; in particular, here *An. coluzzii* is prone to segregate in osmotically more stressful habitats than *An. gambiae*. As human settlements get larger the carrying capacity of the environment increases (*K*_*b*_ > *K*_*a*_); this can potentially shift the stable equilibrium point towards higher densities of *An. gambiae* (e.g. in the smaller urban habitats). Finally, in large and dense urban settlements, where the carrying capacity is at its greatest (*K*_*c*_ > *K*_*b*_), and the larval habitats are osmotically fine-grained (giving less opportunities for the stabilizing effect of habitat selection), the equilibrium point becomes unstable, so that competitive exclusion produces allopatry and competitive release in salinity tolerance (Figs5C and 6B).

### Ecological divergence, phylogeography and speciation

This study provides further evidence of ecological divergence and associated phenotypic differences between *An. coluzzii* and *An. gambiae*. Two novel major environmental gradients along which the species segregate (i.e. distance from the coastline and altitude) have been identified, and their contribution quantitatively integrated in an SDM providing a global map of the predicted relative frequency of *An. coluzzii* and *An. gambiae*. The ‘bimodality’ of the *An. coluzzii* distribution pattern suggests that its coastal and savanna populations may be evolving along independent trajectories. Previous population genetic studies have noted a clear subdivision within *An. coluzzii*, between populations derived from southern coastal Cameroon and populations derived from the West African savannas of Burkina Faso and Mali (Slotman et al. 2007; Lee et al. 2009a). This has led to the view that *An. coluzzii* is structured between West Africa and the Central African rainforest. In accordance with more extensive recent population genetic data (Pinto et al. 2013), our ecological modelling and physiological assays suggest an alternative view that explains the genetic structure equally well: subdivision of *An. coluzzii* is not necessarily between West and Central Africa, but rather between populations occupying the xeric savanna belt in the northern hemisphere, and populations occupying the ribbon of coastline along the Gulf of Guinea (Yawson et al. 2007). If this hypothesis proves correct, it suggests that the region of rampant and apparently recent hybridization between *An. coluzzii* and *An. gambiae* in The Gambia and Guinea Bissau (Caputo et al. 2008, 2011; Oliveira et al. 2008; Marsden et al. 2011; Nwakanma et al. 2013) may represent contact between savanna populations of *An. gambiae* and coastal populations of *An. coluzzii*, not between *An. gambiae* and the better studied northern savanna *An. coluzzii* populations. Only careful reconstruction of the phylogeographic relationships of these taxa across their entire distribution, information sorely lacking at present, will allow this situation to be further clarified.

## Conclusions

Future applications of the quantitative predictive model of habitat segregation and qualitative theoretical framework of species interactions can help address longstanding questions, such as whether ‘mosquitoes select habitats’ or ‘habitats select mosquitoes’ (Harrison 1986; Nosil et al. 2005). Rather than behavioural preference, our data suggest that differences in physiological performance, modulated by environmental-dependent competition, may explain the observed patterns of habitat segregation.

As found in previous studies (Diabate et al. 2008; Lehmann and Diabate 2008; Gimonneau et al. 2010, 2012a,b; Tene Fossog et al. 2013), the ecology of larval stages appears to provide a fertile field of investigation to identify life history traits underlying divergence between *An. coluzzii* and *An. gambiae*. By integrating knowledge about spatially explicit ecological patterns as well as physiological and behavioural responses, we hope to gain a better understanding of the processes that are at the heart of *An. coluzzii* and *An. gambiae* evolution. Ultimately, the aim is to answer questions about how natural selection shapes phenotypic divergence, and what is its role in speciation in this model system. In light of their roles as malignant vectors of malaria, the relevance of evolutionary diversification in this system is amplified by its consequences for disease transmission.

## Data archiving

Data for this study are available at the Dryad Digital Repository: http://doi.org/10.5061/dryad.61m2s.

## Supporting Information

**Figure S1.** Calibration of the minimal adequate SDM mapped in Figure 3A.

**Figure S2.** Coefficients of Variation (CV) of electrical water conductivity (µS/cm) in *Anopheles gambiae s.l*. breeding sites from the forest ecozone in Central Africa.

**Table S1.** Geographical distribution of the breeding sites from which *Anopheles gambiae* and *An. coluzzii* larvae were collected for the salinity tolerance assays.

**Appendix S1.** Statistical analysis of the ecological factors associated with differences in larval salinity tolerance of *Anopheles gambiae s.l*. populations from Central Africa.

## References

[b1] Acevedo P, Ruiz-Fons F, Estrada R, Luz Marquez A, Miranda MA, Gortazar C, Lucientes J (2010). A broad assessment of factors determining *Culicoides imicola* abundance: modelling the present and forecasting its future in climate change scenarios. PLoS ONE.

[b2] Akaike H (1974). A new look at the statistical model identification. IEEE Transactions on Automatic Control.

[b3] Akogbeto M (1995). Entomological study on malaria transmission in coastal and lagoon areas: the case of a village built on a brackish lake. Annales de la Societe Belge de Medecine Tropicale.

[b4] Antonio-Nkondjio C, Fossog BT, Ndo C, Djantio BM, Togouet SZ, Awono-Ambene P, Costantini C (2011). *Anopheles gambiae* distribution and insecticide resistance in the cities of Douala and Yaounde (Cameroon): influence of urban agriculture and pollution. Malaria Journal.

[b5] Awolola TS, Oyewole IO, Amajoh CN, Idowu ET, Ajayi MB, Oduola A, Manafa OU (2005). Distribution of the molecular forms of *Anopheles gambiae* and pyrethroid knock down resistance gene in Nigeria. Acta Tropica.

[b6] Bayoh MN, Thomas CJ, Lindsay SW (2001). Mapping distributions of chromosomal forms of *Anopheles gambiae* in West Africa using climate data. Medical and Veterinary Entomology.

[b7] Bickford D, Lohman DJ, Sodhi NS, Ng PK, Meier R, Winker K, Ingram KK (2007). Cryptic species as a window on diversity and conservation. Trends in Ecology and Evolution.

[b8] Bigoga J, Manga L, Titanji V, Coetzee M, Leke R (2007). Malaria vectors and transmission dynamics in coastal south-western Cameroon. Malaria Journal.

[b9] Bolnick DI, Fitzpatrick BM (2007). Sympatric speciation: models and empirical evidence. Annual Review of Ecology, Evolution, and Systematics.

[b10] Bradley TJ (1987). Physiology of osmoregulation in mosquitoes. Annual Review of Entomology.

[b11] Brown WL, Wilson EO (1956). Character displacement. Systematic Zoology.

[b12] Burnham KP, Anderson DR (2002). Model Selection and Multimodel Inference: A Practical Information-Theoretic Approach.

[b13] Burnham KP, Anderson DR (2004). Multimodel inference: understanding AIC and BIC in model selection. Sociological Methods and Research.

[b14] Calzetta M, Santolamazza F, Carrara GC, Cani PJ, Fortes F, Di Deco MA, della Torre A (2008). Distribution and chromosomal characterization of the *Anopheles gambiae* complex in Angola. The American Journal of Tropical Medicine and Hygiene.

[b15] Caputo B, Nwakanma D, Jawara M, Adiamoh M, Dia I, Konate L, Petrarca V (2008). *Anopheles gambiae* complex along The Gambia river, with particular reference to the molecular forms of *An. gambiae s.s*. Malaria Journal.

[b16] Caputo B, Santolamazza F, Vicente JL, Nwakanma DC, Jawara M, Palsson K, Jaenson T (2011). The “far-west” of *Anopheles gambiae* molecular forms. PLoS ONE.

[b17] Caputo B, Nwakanma D, Caputo FP, Jawara M, Oriero EC, Hamid-Adiamoh M, Dia I (2014). Prominent intra-specific genetic divergence within *Anopheles gambiae* sibling species triggered by habitat discontinuities across a riverine landscape. Molecular Ecology.

[b18] Cassone BJ, Kamdem C, Cheng C, Tan JC, Hahn MW, Costantini C, Besansky NJ (2014). Gene expression divergence between malaria vector sibling species *Anopheles gambiae* and *An. coluzzii* from rural and urban Yaounde Cameroon. Molecular Ecology.

[b19] Cheng C, White BJ, Kamdem C, Mockaitis K, Costantini C, Hahn MW, Besansky NJ (2012). Ecological genomics of *Anopheles gambiae* along a latitudinal cline: a population-resequencing approach. Genetics.

[b20] Coetzee M, Hunt RH, Braack LEO, Davidson G (1993). Distribution of mosquitoes belonging to the *Anopheles gambiae* complex, including malaria vectors, south of latitude 15S. South African Journal of Science.

[b21] Coetzee M, Craig M, le Sueur D (2000). Distribution of African malaria mosquitoes belonging to the *Anopheles gambiae* complex. Parasitology Today.

[b22] Coetzee M, Hunt RH, Wilkerson R, della Torre A, Coulibaly MB, Besansky NJ (2013). *Anopheles coluzzii* and *Anopheles amharicus*, new members of the *Anopheles gambiae* complex. Zootaxa.

[b23] Coluzzi M (1970). Sibling species in *Anopheles* and their importance in malariology. Miscellaneous Publications of the Entomological Society of America.

[b24] Coluzzi M, Barigozzi C (1982). Spatial distribution of chromosomal inversions and speciation in anopheline mosquitoes. Mechanisms of Speciation.

[b25] Coluzzi M, Sabatini A, Petrarca V, Di Deco MA (1979). Chromosomal differentiation and adaptation to human environments in the *Anopheles gambiae* complex. Transactions of the Royal Society of Tropical Medicine and Hygiene.

[b26] Coluzzi M, Sabatini A, Della Torre A, Di Deco MA, Petrarca V (2002). A polytene chromosome analysis of the *Anopheles gambiae* species complex. Science.

[b27] Costantini C, Li SG, Della Torre A, Sagnon N, Coluzzi M, Taylor CE (1996). Density, survival and dispersal of *Anopheles gambiae* complex mosquitoes in a west African Sudan savanna village. Medical and Veterinary Entomology.

[b28] Costantini C, Ayala D, Guelbeogo WM, Pombi M, Some CY, Bassole IHN, Ose K (2009). Living at the edge: biogeographic patterns of habitat segregation conform to speciation by niche expansion in *Anopheles gambiae*. BMC Ecology.

[b29] Coyne JA, Orr HA (2004). Speciation.

[b201] Cuamba N, Choi K, Townson H (2006). Malaria vectors in Angola: distribution of species and molecular forms of the Anopheles gambiae complex, their pyrethroid insecticide knockdown resistance (kdr) status and Plasmodium falciparum sporozoite rates. Malaria Journal.

[b30] Dabire RK, Namountougou M, Sawadogo SP, Yaro LB, Toe HK, Ouari A, Gouagna LC (2012). Population dynamics of *Anopheles gambiae s.l*. in Bobo-Dioulasso city: bionomics, infection rate and susceptibility to insecticides. Parasites and Vectors.

[b31] Dabire KR, Sawadodgo S, Diabate A, Toe KH, Kengne P, Ouari A, Costantini C (2013). Assortative mating in mixed swarms of the mosquito *Anopheles gambiae s.s*. M and S molecular forms, in Burkina Faso, West Africa. Medical and Veterinary Entomology.

[b32] Deitz KC, Athrey G, Reddy MR, Overgaard HJ, Matias A, Jawara M, Della Torre A (2012). Genetic isolation within the malaria mosquito *Anopheles melas*. Molecular Ecology.

[b34] Diabate A, Dabire RK, Kim EH, Dalton R, Millogo N, Baldet T, Simard F (2005). Larval development of the molecular forms of *Anopheles gambiae* (Diptera: Culicidae) in different habitats: a transplantation experiment. Journal of Medical Entomology.

[b35] Diabate A, Dabire RK, Kengne P, Brengues C, Baldet T, Ouari A, Simard F (2006). Mixed swarms of the molecular M and S forms of *Anopheles gambiae* (Diptera: Culicidae) in sympatric area from Burkina Faso. Journal of Medical Entomology.

[b36] Diabate A, Dabire RK, Millogo N, Lehmann T (2007). Evaluating the effect of postmating isolation between molecular forms of *Anopheles gambiae* (Diptera: Culicidae). Journal of Medical Entomology.

[b37] Diabate A, Dabire KR, Heidenberger K, Crawford J, Lamp W, Culler L, Lehmann T (2008). Evidence for divergent selection between the molecular forms of *Anopheles gambiae*: role of predation. BMC Evolutionary Biology.

[b38] Diabate A, Dao A, Yaro AS, Adamou A, Gonzalez R, Manoukis NC, Traore SF (2009). Spatial swarm segregation and reproductive isolation between the molecular forms of *Anopheles gambiae*. Proceedings of the Royal Society of London. Series B: Biological Sciences.

[b39] Dieckmann U, Metz JAJ, Doebeli M, Tautz D (2004). Adaptive Speciation.

[b40] Djogbenou L, Pasteur N, Akogbeto M, Weill M, Chandre F (2011). Insecticide resistance in the *Anopheles gambiae* complex in Benin: a nationwide survey. Medical and Veterinary Entomology.

[b41] Fanello C, Santolamazza F, della Torre A (2002). Simultaneous identification of species and molecular forms of the *Anopheles gambiae* complex by PCR-RFLP. Medical and Veterinary Entomology.

[b42] Ferguson HM, Dornhaus A, Beeche A, Borgemeister C, Gottlieb M, Mulla MS, Gimnig JE (2010). Ecology: a prerequisite for malaria elimination and eradication. PLoS Medicine.

[b43] Gillies MT, Coetzee M (1987). A Supplement to the Anophelinae of Africa South of the Sahara.

[b44] Gillies MT, De Meillon B (1968). The Anophelinae of Africa South of the Sahara.

[b45] Gimonneau G, Bouyer J, Morand S, Besansky NJ, Diabate A, Simard F (2010). A behavioral mechanism underlying ecological divergence in the malaria mosquito *Anopheles gambiae*. Behavioral Ecology.

[b46] Gimonneau G, Pombi M, Choisy M, Morand S, Dabire RK, Simard F (2012a). Larval habitat segregation between the molecular forms of the mosquito, *Anopheles gambiae* in a rice field area of Burkina Faso, West Africa. Medical and Veterinary Entomology.

[b47] Gimonneau G, Pombi M, Dabire RK, Diabate A, Morand S, Simard F (2012b). Behavioural responses of *Anopheles gambiae sensu stricto* M and S molecular form larvae to an aquatic predator in Burkina Faso. Parasites and Vectors.

[b48] Guisan A, Zimmerman NE (2000). Predictive habitat distribution models in ecology. Ecological Modelling.

[b49] Hahn MW, White BJ, Muir CJ, Besansky NJ (2012). No evidence for biased co-transmission of speciation islands in *Anopheles gambiae*. Philosophical Transactions of the Royal Society of London. Series B: Biological Sciences.

[b50] Harbach RE, Manguin S (2013). The phylogeny and classification of *Anopheles*. Anopheles Mosquitoes – New Insights into Malaria Vectors.

[b51] Harrison RG (1986). Pattern and process in a narrow hybrid zone. Heredity.

[b52] Hijmans RJ, Cameron SE, Parra JL, Jones PG, Jarvis A (2005). Very high resolution interpolated climate surfaces for global land areas. International Journal of Climatology.

[b53] Hosmer DW, Lemeshow S (2000). Applied logistic regression.

[b54] Jovani R, Tella JL (2006). Parasite prevalence and sample size: misconceptions and solutions. Trends in Parasitology.

[b55] Kamdem C, Tene Fossog B, Simard F, Etouna J, Ndo C, Kengne P, Bousses P (2012). Anthropogenic habitat disturbance and ecological divergence between incipient species of the malaria mosquito *Anopheles gambiae*. PLoS ONE.

[b56] Lee Y, Cornel AJ, Meneses CR, Fofana A, Andrianarivo AG, McAbee RD, Fondjo E (2009a). Ecological and genetic relationships of the Forest-M form among chromosomal and molecular forms of the malaria vector *Anopheles gambiae sensu stricto*. Malaria Journal.

[b57] Lee Y, Meneses CR, Fofana A, Lanzaro GC (2009b). Desiccation resistance among subpopulations of *Anopheles gambiae s.s*. from Selinkenyi, Mali. Journal of Medical Entomology.

[b58] Legendre P, Legendre L (1998). Numerical Ecology.

[b59] Lehmann T, Diabate A (2008). The molecular forms of *Anopheles gambiae*: a phenotypic perspective. Infection, Genetics and Evolution.

[b60] Levine RS, Peterson AT, Benedict MQ (2004). Geographic and ecologic distributions of the *Anopheles gambiae* complex predicted using a genetic algorithm. American Journal of Tropical Medicine and Hygiene.

[b61] Lindsay SW, Parson L, Thomas CJ (1998). Mapping the ranges and relative abundance of the two principal African malaria vectors, *Anopheles gambiae sensu stricto* and *An. arabiensis*, using climate data. Proceedings of the Royal Society of London. Series B: Biological Sciences.

[b62] Losos JB (2000). Ecological character displacement and the study of adaptation. Proceedings of the National Academy of Sciences of the United States of America.

[b63] Losos JB, Leal M, Glor RE, de Queiroz K, Hertz PE, Schettino LR, Lara AC (2003). Niche lability in the evolution of a Caribbean lizard community. Nature.

[b64] Manoukis NC, Powell JR, Touré MB, Sacko A, Edillo FE, Coulibaly MB, Traoré SF (2008). A test of the chromosomal theory of ecotypic speciation in *Anopheles gambiae*. Proceedings of the National Academy of Sciences of the United States of America.

[b65] Marsden C, Lee Y, Neimen C, Sanford M, Dinis J, Martins C, Rodrigues A (2011). Asymmetric introgression between the M and S molecular forms of the malaria vector, *Anopheles gambiae*, maintains divergence despite extensive hybridisation. Molecular Ecology.

[b66] Martin RA, Pfennig DW (2011). Evaluating the targets of selection during character displacement. Evolution.

[b67] Masendu HT, Hunt RH, Govere J, Brooke BD, Awolola TS, Coetzee M (2004). The sympatric occurrence of two molecular forms of the malaria vector *Anopheles gambiae* Giles *sensu stricto* in Kanyemba, in the Zambezi Valley, Zimbabwe. Transactions of the Royal Society of Tropical Medicine and Hygiene.

[b68] Miller ME, Hui SL, Tierney WM (1991). Validation techniques for logistic-regression models. Statistics in Medicine.

[b69] Moffett A, Shackelford N, Sarkar S (2007). Malaria in Africa: Vector Species’ Niche Models and Relative Risk Maps. PLoS ONE.

[b70] Morlais I, Poncon N, Simard F, Cohuet A, Fontenille D (2004). Intraspecific nucleotide variation in *Anopheles gambiae*: new insights into the biology of malaria vectors. The American Journal of Tropical Medicine and Hygiene.

[b71] Mosha FW, Mutero CM (1982). The influence of salinity on larval development and population dynamics of *Anopheles merus* Dönitz (Diptera: Culicidae). Bulletin of Entomological Research.

[b72] Ndiath MO, Cohuet A, Gaye A, Konate L, Mazenot C, Faye O, Boudin C (2011). Comparative susceptibility to *Plasmodium falciparum* of the molecular forms M and S of *Anopheles gambiae* and *Anopheles arabiensis*. Malaria Journal.

[b73] Neafsey DE, Lawniczak MK, Park DJ, Redmond SN, Coulibaly MB, Traore SF, Sagnon N (2010). SNP genotyping defines complex gene-flow boundaries among African malaria vector mosquitoes. Science.

[b74] Nicholson SE, Farrar TJ (1994). The influence of soil type on the relationships between NDVI, rainfall, and soil-moisture in semiarid Botswana. 1. NDVI response to rainfall. Remote Sensing of the Environment.

[b75] Nicholson SE, Davenport ML, Malo AR (1990). A comparison of the vegetation response to rainfall in the Sahel and East Africa, using normalized difference vegetation index from NOAA AVHRR. Climatic Change.

[b76] Nosil P (2012). Ecological Speciation.

[b77] Nosil P, Vines TH, Funk DJ (2005). Perspective: reproductive isolation caused by natural selection against immigrants from divergent habitats. Evolution.

[b78] Nwakanma DC, Neafsey DE, Jawara M, Adiamoh M, Lund E, Rodrigues A, Loua KM (2013). Breakdown in the process of incipient speciation in *Anopheles gambiae*. Genetics.

[b79] Oliveira E, Salgueiro P, Palsson K, Vicente JL, Arez AP, Jaenson TG, Caccone A (2008). High levels of hybridization between molecular forms of *Anopheles gambiae* from Guinea Bissau. Journal of Medical Entomology.

[b80] Olson DM (2001). Terrestrial ecoregions of the worlds: a new map of life on Earth. BioScience.

[b81] Orr MR, Smith TB (1998). Ecology and speciation. Trends in Ecology and Evolution.

[b82] Pearce J, Ferrier S (2000). Evaluating the predictive performance of habitat models developed using logistic regression. Ecological Modelling.

[b83] Pennetier C, Warren B, Dabire KR, Russell IJ, Gibson G (2010). “Singing on the wing” as a mechanism for species recognition in the malarial mosquito *Anopheles gambiae*. Current Biology.

[b84] Peterson AT (2006). Ecologic niche modeling and spatial patterns of disease transmission. Emerging Infectious Diseases.

[b85] Peterson AT, Soberon J, Sanchez-Cordero V (1999). Conservatism of ecological niches in evolutionary time. Science.

[b86] Pfenninger M, Schwenk K (2007). Cryptic animal species are homogeneously distributed among taxa and biogeographical regions. BMC Evolutionary Biology.

[b87] Pinto J, Egyir-Yawson A, Vicente J, Gomes B, Santolamazza F, Moreno M, Charlwood J (2013). Geographic population structure of the African malaria vector *Anopheles gambiae* suggests a role for the forest-savannah biome transition as a barrier to gene flow. Evolutionary Applications.

[b202] Rosenzweig ML (1981). A theory of habitat selection. Ecology.

[b88] Reidenbach KR, Neafsey DE, Costantini C, Sagnon N'F, Simard F, Ragland GJ, Egan SP (2012). Patterns of genomic differentiation between ecologically differentiated M and S forms of *Anopheles gambiae* in West and Central Africa. Genome Biology and Evolution.

[b89] Rice AM, Pfennig DW (2010). Does character displacement initiate speciation? Evidence of reduced gene flow between populations experiencing divergent selection. Journal of Evolutionary Biology.

[b90] Ridl F, Bass C, Torrez M, Govender D, Ramdeen V, Yellot L, Edu A (2008). A pre-intervention study of malaria vector abundance in Rio Muni, Equatorial Guinea: Their role in malaria transmission and the incidence of insecticide resistance alleles. Malaria Journal.

[b91] Santolamazza F, della Torre A, Caccone A (2004). Short report: a new polymerase chain reaction-restriction fragment length polymorphism method to identify *Anopheles arabiensis* from *An. gambiae* and its two molecular forms from degraded DNA templates or museum samples. American Journal of Tropical Medicine and Hygiene.

[b92] Santolamazza F, Calzetta M, Etang J, Barrese E, Dia I, Caccone A, Donnelly MJ (2008). Distribution of knock-down resistance mutations in *Anopheles gambiae* molecular forms in west and west-central Africa. Malaria Journal.

[b93] Sawadogo PS, Namountougou M, Toe KH, Rouamba J, Maiga H, Ouedraogo KR, Baldet T (2014). Swarming behaviour in natural populations of *Anopheles gambiae* and *An. coluzzii*: review of 4 years survey in rural areas of sympatry, Burkina Faso (West Africa). Acta Tropica.

[b94] Schluter D (2000). The Ecology of Adaptive Radiation.

[b95] Schluter D, McPhail JD (1992). Ecological character displacement and speciation in sticklebacks. American Naturalist.

[b96] Schluter D, McPhail JD (1993). Character displacement and replicate adaptive radiation. Trends in Ecology & Evolution.

[b97] Scott JA, Brogdon WG, Collins FH (1993). Identification of single specimens of the *Anopheles gambiae* complex by the polymerase chain reaction. American Journal of Tropical Medicine and Hygiene.

[b98] Service MW (1993). Mosquito Ecology. Field Sampling Methods.

[b99] Simard F, Ayala D, Kamdem GC, Etouna J, Ose K, Fotsing J-M, Fontenille D (2009). Ecological niche partitioning between the M and S molecular forms of *Anopheles gambiae* in Cameroon: the ecological side of speciation. BMC Ecology.

[b100] Sinka ME, Bangs MJ, Manguin S, Coetzee M, Mbogo CM, Hemingway J, Patil AP (2010). The dominant *Anopheles* vectors of human malaria in Africa, Europe and the Middle East: occurrence data, distribution maps and bionomic precis. Parasites and Vectors.

[b101] Slotman MA, Tripet F, Cornel AJ, Meneses CR, Lee Y, Reimer LJ, Thiemann TC (2007). Evidence for subdivision within the M molecular form of *Anopheles gambiae*. Molecular Ecology.

[b102] Sobel JM, Chen GF, Watt LR, Schemske DW (2010). The biology of speciation. Evolution.

[b103] de Souza D, Kelly-Hope L, Lawson B, Wilson M, Boakye D (2010). Environmental factors associated with the distribution of *Anopheles gambiae s.s*. in Ghana; an important vector of lymphatic filariasis and malaria. PLoS ONE.

[b104] Szilagyi A, Meszena G (2009). Limiting similarity and niche theory for structured populations. Journal of Theoretical Biology.

[b105] Tanga MC, Ngundu WI, Judith N, Mbuh J, Tendongfor N, Simard F, Wanji S (2010). Climate change and altitudinal structuring of malaria vectors in south-western Cameroon: their relation to malaria transmission. Transactions of the Royal Society of Tropical Medicine and Hygiene.

[b106] Tene Fossog B, Antonio-Nkondjio C, Kengne P, Njiokou F, Besansky NJ, Costantini C (2013). Physiological correlates of ecological divergence along an urbanization gradient: differential tolerance to ammonia among molecular forms of the malaria mosquito *Anopheles gambiae*. BMC Ecology.

[b33] della Torre A, Tu Z, Petrarca V (2005). On the distribution and genetic differentiation of Anopheles gambiae s.s. molecular forms. Insect Biochemistry and Molecular Biology.

[b107] Tripet F, Toure YT, Taylor CE, Norris DE, Dolo G, Lanzaro GC (2001). DNA analysis of transferred sperm reveals significant levels of gene flow between molecular forms of *Anopheles gambiae*. Molecular Ecology.

[b108] Turelli M, Barton NH, Coyne JA (2001). Theory and speciation. Trends in Ecology and Evolution.

[b203] Turner RE, Rabalais NN (1994). Coastal eutrophication near the Mississippi river delta. Nature.

[b109] Tyerman J, Bertrand M, Spencer C, Doebeli M (2008). Experimental demonstration of ecological character displacement. BMC Evolutionary Biology.

[b110] Vezenegho SB, Brooke BD, Hunt RH, Coetzee M, Koekemoer LL (2009). Malaria vector composition and insecticide susceptibility status in Guinea Conakry, West Africa. Medical and Veterinary Entomology.

[b111] White BJ, Lawniczak MK, Cheng C, Coulibaly MB, Wilson MD, Sagnon N, Costantini C (2011a). Adaptive divergence between incipient species of *Anopheles gambiae* increases resistance to *Plasmodium*. Proceedings of the National Academy of Sciences of the United States of America.

[b112] White BJ, Collins FH, Besansky NJ (2011b). Evolution of *Anopheles gambiae* in relation to humans and malaria. Annual Review in Ecology, Evolution, and Systematics.

[b113] Yawson AE, Weetman D, Wilson MD, Donnelly MJ (2007). Ecological zones rather than molecular forms predict genetic differentiation in the malaria vector *Anopheles gambiae s.s*. in Ghana. Genetics.

[b114] Zheng BY, Agresti A (2000). Summarizing the predictive power of a generalized linear model. Statistics in Medicine.

