# Supplementary figures and images for: Habitat segregation and ecological character displacement in cryptic African malaria mosquitoes
Source: Evol Appl. 2015 Mar 8;8(4):326–45. doi: 10.1111/eva.12242 (PMC4408144; doi:10.1111/eva.12242)

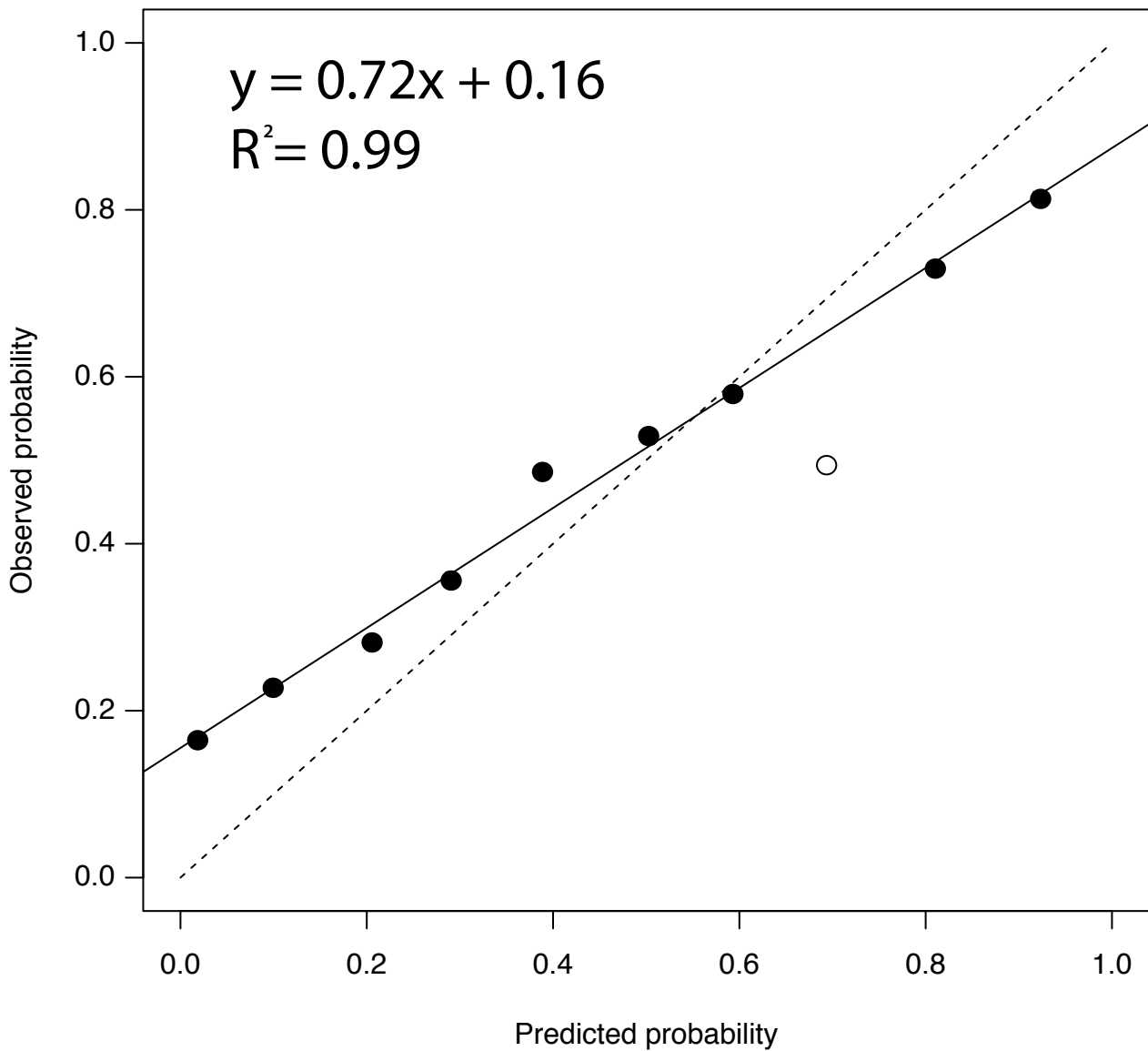

Supplement: Supplementary file 1 [file eva0008-0326-sd1.pdf]

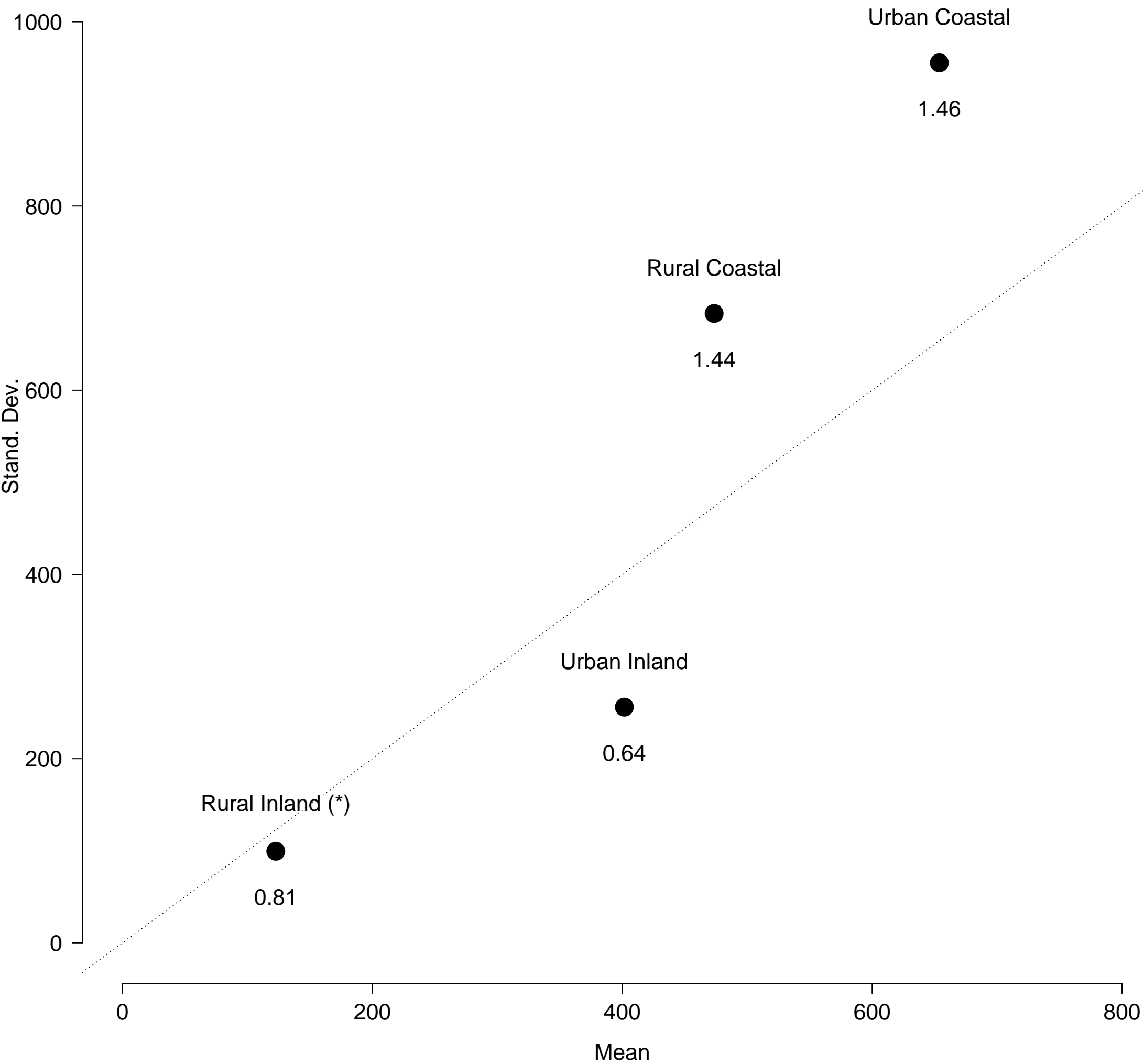

Supplement: Supplementary file 2 [file eva0008-0326-sd2.pdf]
