# Supplementary material for: Habitat segregation and ecological character displacement in cryptic African malaria mosquitoes
Source: Evol Appl. 2015 Mar 8;8(4):326–45. doi: 10.1111/eva.12242 (PMC4408144; doi:10.1111/eva.12242)

ECOLOGICAL FACTORS ASSOCIATED WITH DIFFERENCES IN LARVAL SALINITY TOLERANCE OF *ANOPHELES GAMBIAE S.L.* POPULATIONS FROM CENTRAL AFRICA

Supplementary material from:

“Habitat segregation and ecological character displacement in cryptic African malaria mosquitoes”

Billy Tene Fossog, Diego Ayala, Pelayo Acevedo, Pierre Kengne, Ignacio Ngomo Abeso Mebuy, Boris Makanga, Julie Magnus, Parfait Awono-Ambene, Flobert Njiokou, Marco Pombi, Christophe Antonio-Nkondjio, Christophe Paupy, Nora J. Besansky, Carlo Costantini

We fitted binomial GLMMs to the whole bioassay dataset (Supplementary Table S1) without taking into account which taxon among *Anopheles coluzzii* and *An. gambiae* was being exposed. Accordingly, all tested larvae from breeding sites returning no mortality in the control were included in this global analysis. Three variables were considered in the full model, the “Salinity concentration” covariable, the “Habitat” and “Location” factors, and their 2nd and 3rd order interactions (model No. 128 in Table A below). Other competitive models were tested by taking out each one of these variables in turn (or combinations thereof), as well as their interactions (Table A). Three models had substantial statistical support based on the ΔAIC_c and Akaike weights criteria. All three included at least “Salinity concentration”—as expected—and the “Habitat” factor, indicating these were the major predictor variables. The best model (No. 32 in Table A, i.e. that having $\Delta AIC_c = 0$) included also the “Location” factor and two interactions “Habitat x Location”, and “Habitat x Salinity”, indicating that populations had a similar response to “Salinity concentration” (i.e. slope of the logistic regression lines) within the same location but different responses across habitat types, and different intercepts according to “Habitat” or “Location”, indicating a different degree of tolerance in populations living in different locations and habitat types. The second-and third-best models had the same degree of statistical support, and were only marginally worse than the best one (Akaike weights: 0.123 *vs*. 0.300, respectively); the third-best model (No. 6 in Table A) did not include the “Location” factor nor any interaction with “Habitat”, indicating that differences in salinity tolerance were mainly accounted for by the urban or rural nature of the environment rather than by distance from the coast. The second-best model was similar to the best one except that the interaction term “Habitat x Salinity” was replaced by “Location x Salinity”. Overall, the relative importance of the fixed effects was “Salinity”=1, “Habitat”=1, “Location”=0.77, “Habitat x Location”=0.77, “Habitat x Salinity”=0.55, and “Location x Salinity”=0.23. The ensuing mortality dose-response curves drawn from multi-model parameter estimates show that urban populations were more tolerant to salinity than rural populations (Figure A below). However, by comparing locations within each habitat type, somewhat unexpectedly coastal populations were not necessarily more tolerant than inland ones (Figure A).

Table A. Evaluation of competitive GLMMs fitted to the salinity tolerance assays data. Fixed effects terms included in the full model (Model No. 128) are the factors "Habitat" (Hbt, two levels: rural *vs.* urban); "Location" (Loc, two levels: coastal *vs.* inland); and the covariable "Salinity concentration" (Sty, expressed as a percentage dilution of seawater); plus their second- and third-order interactions. The intercept (logit scale) refers to the fitted value for baseline factor levels and the covariable set to zero. Random effects are salinity grouped by breeding sites nested within localities. The dotted line separates models with greater statistical support, i.e. those whose $\Delta AIC_c \leq 2$.

Model No. (Rank)	Model Terms (Fixed Effects)								d.f.	ΔAIC_c	Akaike Weight
	Intercept	Salinity	Hbt	Loc	Hbt × Loc	Hbt × Sty	Loc × Sty	Hbt × Loc × Sty			
32 (01)	-9.401	0.3161	+	+	+	+			12	0	0.300
48 (02)	-8.381	0.2840	+	+	+		+		12	1.78	0.123
6 (03)	-7.831	0.2833	+						9	1.79	0.123
22 (04)	-8.818	0.3151	+			+			10	2.16	0.102
16 (05)	-8.387	0.2825	+	+	+				11	2.30	0.095
8 (06)	-7.684	0.2831	+	+					10	2.87	0.071
24 (07)	-8.648	0.3141	+	+		+			11	3.34	0.057
128 (08)	-9.186	0.3093	+	+	+	+	+	+	14	4.02	0.040
64 (09)	-9.448	0.3169	+	+	+	+	+		13	4.50	0.032
40 (10)	-7.741	0.2849	+	+			+		11	4.91	0.026
56 (11)	-8.678	0.3150	+	+		+	+		12	5.40	0.020
5 (12)	-9.008	0.2835							8	7.71	0.006
7 (13)	-8.723	0.2833		+					9	8.04	0.005
39 (14)	-8.829	0.2866		+			+		10	13.59	0
1 (15)	-3.859	0							7	266.4	0
3 (16)	-2.664	0		+					8	295.7	0
4 (17)	-1.725	0	+	+					9	338.9	0
12 (18)	-1.703	0	+	+	+				10	348.8	0
2 (19)	-2.272	0	+						8	366.3	0

Figure A. Dose-mortality response to salinity in larvae of *An. gambiae* and *An. coluzzii* from the Central African rainforest ecozone. Observed proportions (points: coastal; squares: inland) of dead larvae after 24 h exposure to salt whose concentration is expressed as percent of seawater (100% = 35 g L^{-1}). Fitted logistic curves for different habitats (green: rural; white: urban) and locations (solid line: coastal; dashed line: inland) are drawn from model-averaged parameters extracted from the three models having substantial statistical support in Table A.

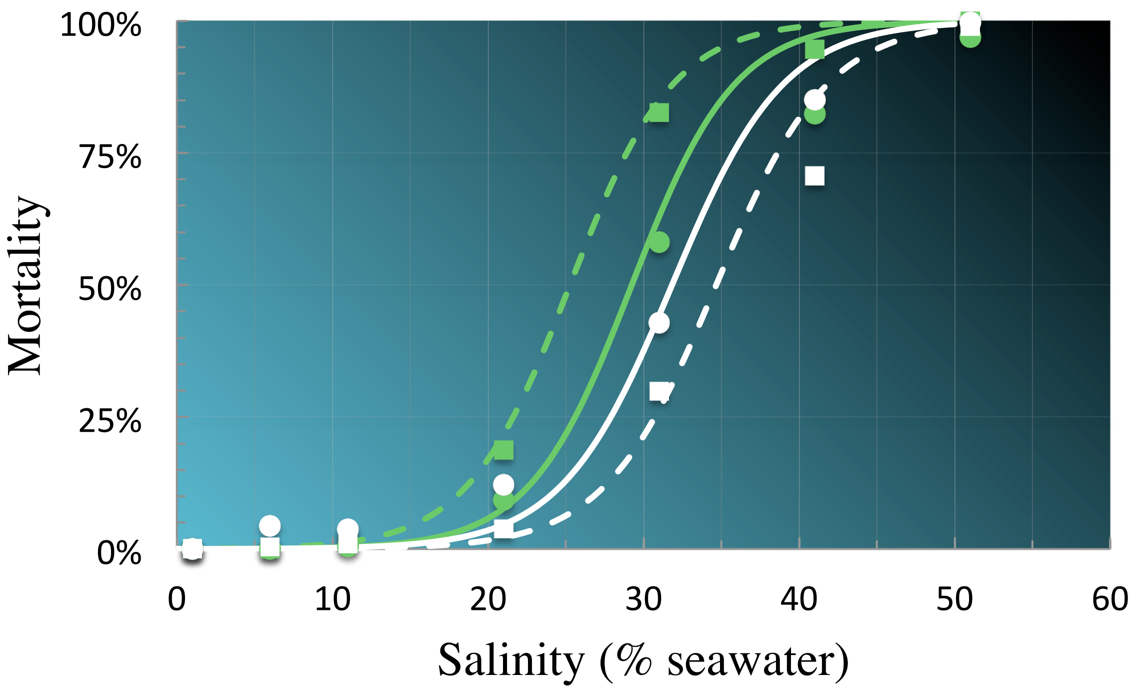

Supplement: Supplementary file 4 [file eva0008-0326-sd4.pdf]
